# Inhibitor of Apoptosis-Stimulating Protein of p53 (iASPP) Is Required for Neuronal Survival after Axonal Injury

**DOI:** 10.1371/journal.pone.0094175

**Published:** 2014-04-08

**Authors:** Ariel M. Wilson, Vince A. Chiodo, Sanford L. Boye, Nicholas C. Brecha, William W. Hauswirth, Adriana Di Polo

**Affiliations:** 1 Department of Neuroscience and Groupe de Recherche sur le Système Nerveux Central, University of Montreal Hospital Research Center (CR-CHUM), University of Montreal, Montreal, Quebec, Canada; 2 Department of Ophthalmology, College of Medicine, University of Florida, Gainesville, Florida, United States of America; 3 Departments of Neurobiology and Medicine, David Geffen School of Medicine at University of California Los Angeles, Los Angeles, California, United States of America; Duke University, United States of America

## Abstract

The transcription factor p53 mediates the apoptosis of post-mitotic neurons exposed to a wide range of stress stimuli. The apoptotic activity of p53 is tightly regulated by the apoptosis-stimulating proteins of p53 (ASPP) family members: ASPP1, ASPP2 and iASPP. We previously showed that the pro-apoptotic members ASPP1 and ASPP2 contribute to p53-dependent death of retinal ganglion cells (RGCs). However, the role of the p53 inhibitor iASPP in the central nervous system (CNS) remains to be elucidated. To address this, we asked whether iASPP contributes to the survival of RGCs in an *in vivo* model of acute optic nerve damage. We demonstrate that iASPP is expressed by injured RGCs and that iASPP phosphorylation at serine residues, which increase iASPP affinity towards p53, is significantly reduced following axotomy. We show that short interference RNA (siRNA)-induced iASPP knockdown exacerbates RGC death, whereas adeno-associated virus (AAV)-mediated iASPP expression promotes RGC survival. Importantly, our data also demonstrate that increasing iASPP expression in RGCs downregulates p53 activity and blocks the expression of pro-apoptotic targets PUMA and Fas/CD95. This study demonstrates a novel role for iASPP in the survival of RGCs, and provides further evidence of the importance of the ASPP family in the regulation of neuronal loss after axonal injury.

## Introduction

iASPP is the most evolutionarily conserved member of the ‘Ankyrin-repeat, SH3-domain, and Proline-rich-region containing Protein’ (ASPP) family [1], comprised of ASPP1, ASPP2, and iASPP. The first detected form of iASPP, a truncated variant termed RelA-associated inhibitor (RAI), was identified as a nuclear factor kappa beta (NFκB) inhibitor in a yeast two hybrid screen [2]. The full-length isoform of iASPP, which is the predominant form of this molecule expressed in cells, was later discovered and shown to carry a C-terminus identical to RAI [3]. ASPP family members have attracted much attention since their implication in a novel mechanism of p53 apoptotic regulation was identified in cancer cells. During tumorigenesis, pro-apoptotic ASPP1/2 enhance p53-dependent cell death [4]–[7], while anti-apoptotic iASPP binds to p53 to inhibit its ability to transactivate pro-apoptotic target genes [5], [7]–[11].

Since its discovery, iASPP was shown to be encoded by the Protein Phosphatase 1 Regulatory Subunit 13-Like (PPP1R13L) gene, which is overexpressed in many tumors including acute leukemia [12], breast cancer [1], glioblastoma [13], ovarian cancer [14], and head and neck squamous cell carcinoma [15]. Previous studies demonstrated that overexpression of iASPP in a human osteosarcoma cell line increased their resistance to ultraviolet radiation or cisplatin-induced apoptosis, without altering p53 expression [1]. Due to its potent inhibitory role of p53 apoptotic activity, iASPP function has been studied primarily in cancer cells or in the context of tumor biology. However, the role of iASPP in neuronal survival and neurodegeneration is not well understood.

To address this, we asked whether iASPP is implicated in the survival of retinal ganglion cells (RGC) after axonal injury. RGCs are central nervous system (CNS) neurons that undergo a predictable onset of apoptotic death following optic nerve transection [16], [17]. Here, we demonstrate that iASPP is expressed by adult intact and axotomized RGCs. We show that short interference RNA (siRNA)-mediated knockdown of retinal iASPP expression exacerbates RGC death, while iASPP overexpression using serotype 2 adeno-associated virus (AAV) promotes RGC survival *in vivo*. We demonstrate that increased iASPP expression leads to a reduction in p53 apoptotic activity as evidenced by downregulation of p53 phosphoserine 15 (pSer15 p53), and its targets PUMA and Fas/CD95. In summary, our study identifies a novel role for iASPP in the survival of RGCs after acute optic nerve damage, and further supports a critical role of ASPP family members in the regulation of neuronal loss in the injured CNS.

## Materials and Methods

### Experimental Animals

Animal procedures were performed in accordance with the guidelines of the Canadian Council on Animal Care for the use of experimental animals (www.ccac.ca). All protocols were approved by the Committee on the Ethics of Use of Experimental Animals at the University of Montreal (Permit Number: 13-018). Surgeries were carried out in adult, female Sprague-Dawley rats (180–200 g), and performed under general anesthesia (2% Isoflurane, 0.8 liter/min). The number of animals used in each experiment is indicated in the results and the legend of the corresponding figure. All efforts were made to minimize the suffering of experimental animals.

### Optic nerve axotomy

The left optic nerve was exposed and carefully transected at 1 mm from the optic nerve head avoiding injury to the ophthalmic artery, as previously described [17]–[19]. The right eye was never operated on and served as internal control. Fundus examination was performed immediately after axotomy and three days later to check the integrity of the retinal circulation after surgery. Animals showing signs of compromised blood supply were excluded from the study.

### Short interfering RNA (siRNA)

The siRNA sequences against iASPP were purchased from Dharmacon (Smartpool, Thermo Scientific, Lafayette, CO) (sense strands): 5′-CCGCCAA AGUGGACGAAUU-3′, 5′-UGACAGGCGGUUCUGACGUU-3′, 5′-CCGAAGGCCU GGAACGAGU-3′, 5′-UGGUACAGCAGGCGGUGAA-3′. The control siRNA against GFP was kindly provided by Dr. Elena Feinstein (Quark Pharmaceuticals Inc.) and has been described elsewhere [20].

### Recombinant AAV Serotype 2 Vectors

A murine iASPP cDNA containing a c-terminal, myc tag (synthesized by GenScript USA Inc., Piscataway, NJ) was inserted downstream of the Synapsin 1 (Syn1) promoter into an AAV vector plasmid containing bovine growth hormone poly A and AAV serotype 2 terminal repeats. Site-directed mutagenesis of surface-exposed tyrosine residues on AAV2 was done to prevent proteasome-mediated degradation and improve transduction efficiency as previously described [21]. Vectors were packaged, concentrated, and titered using standard methods [22]. A control vector containing the green fluorescent protein (GFP) gene under control of the same Syn1 promoter was prepared in identical fashion and used as control. The titers of the vector stocks were: 1.07E+13 vector genomes/ml (vg/ml) for hSyn1-iASPP-myc AAV2 Triple Y-F (AAV.iASPP) and 8.23E+12 vg/ml for Syn1-hGFP AAV2 Triple Y-F (AAV.GFP).

### Intravitreal injections

siRNA against iASPP or control siGFP (2 μg/μl), as well as AAV encoding iASPP (AAV.iASPP, 1.07E+13 vg/ml) or control GFP (AAV.GFP, 8.23E+12 vg/ml) were injected into the vitreous chamber of the left eye using a Hamilton syringe fitted with a 32-gauge glass microneedle (total volume: 5 μl). The sclera was exposed and the tip of the needle inserted at a 45° angle through the sclera and retina into the vitreous space using a posterior approach. This route of administration avoided injury to anterior eye structures, which can promote RGC survival [23], [24]. Surgical glue (Indermill, Tyco Health Care, Mansfield, MA) was used to seal the injection site. Intraocular injection of siRNA was performed at the time of optic nerve axotomy, while injection of AAV was performed two weeks prior to axotomy to allow for AAV-mediated transgene expression to reach a plateau [17], [19].

### Retinal immunohistochemistry

Animals were perfused transcardially with 4% paraformaldehyde (PFA) in 0.1 M phosphate buffered saline (PBS, pH 7.4). Retinal cryosections (16 μm) were prepared as previously described [19], [25]. For iASPP immunohistochemistry, retinas were subjected to heat-mediated antigen retrieval by incubating sections in 0.01 M sodium citrate in 0.5% Tween-20 (pH 6) at 85°C for 15 min. The following primary antibodies were added to the retinal sections in blocking solution and incubated overnight at 4°C: RNA binding protein with multiple splicing (RBPMS), Brn3a (1 μg/ml, Santa Cruz Biotechnologies, Santa Cruz, CA), iASPP (1 μg/ml, Bethyl Laboratories, Montgomery, TX), iASPP (1 μg/mL, Clone LXO49.3, 1 μg/ml, Sigma-Aldrich, Saint-Louis, MO), Calretinin (1∶1000, Millipore, Billerica, MA), Calbindin (1∶10,000, Swant, Switzerland), or c-myc (1 μg/mL, Abcam, Cambridge, MA). The selective RGC markers RBPMS and Brn3a were used for distinct purposes: RBPMS was used for cytoplasmic co-localization studies, whereas nuclear Brn3a was used for RGC density quantification. For RBPMS, rabbit and guinea pig polyclonal antibodies were generated against the N-terminus GGKAEKENTPSEANLQEEEVR (RBPMS_4-24_) by ProSci Inc. (Powy, CA). Sera were collected following immunization and affinity purified using a RBPMS polypeptide affinity column as described [26]. Sections were then incubated with secondary antibodies: anti-rabbit IgG, anti-mouse IgG, or anti-goat IgG (1–8 μg/ml, Cy3, Alexa 594, Alexa 488, Alexa 350, Jackson ImmunoResearch Laboratories Inc., West Grove, PA), washed and mounted in anti-fade reagent (SlowFade, Molecular Probes, Eugene, OR). Fluorescent labeling was observed with a Zeiss AxioSkop 2 Plus microscope (Carl Zeiss Canada, Kirkland, QC).

### Western blot analysis

Whole fresh retinas were rapidly dissected and homogenized with an electric pestle (Kontes, Vineland, NJ) in ice-cold lysis buffer (20 mM Tris pH 8.0, 135 mM NaCl, 1% NP-40, 0.1% SDS, and 10% glycerol supplemented with protease inhibitors). For phosphorylated protein analysis, retinas were homogenized in ice-cold phosphorylation lysis buffer (50 mM Tris HCl pH 7.4, EDTA 1 mM, NaCl 150 mM, NP40 1%, NaF 5 mM, Na deoxycholate 0.25%, NaVO_3_ 2 mM, supplemented with protease and phosphatase inhibitors). Protein homogenates were centrifuged at 10,000 rpm for 10 min, and the supernatants removed and resedimented for an additional 10 min to yield solubilized extracts. Retinal extracts (40 μg) were resolved on SDS polyacrylamide gels and transferred to nitrocellulose membranes (Bio-Rad Life Science, Mississauga, ON). Blots were incubated overnight at 4°C with each of the following primary antibodies: phospho-p53 (Ser15) (2 μg/ml, Abcam), acetyl p53 (Lys 373, Lys 382) (1∶100, Millipore), iASPP (0.5 μg/ml, Bethyl Laboratories), iASPP (0.1 μg/ml, Clone LXO49.3, Sigma-Aldrich), Bax (1.5 μg/ml, N20, Santa Cruz Biotechnologies), PUMA (1 μg/ml, Abcam), Noxa (0.5 μg/ml, Sigma-Aldrich), Fas/CD95 (1 μg/ml, BD Transduction Laboratories, San Jose, CA) or β-actin (0.5 μg/ml, Sigma-Aldrich). Membranes were washed and incubated in anti-rabbit or anti-mouse peroxidase-linked secondary antibodies (0.5 μg/ml, Amersham Biosciences, Baie d'Urfé, QC). Blots were developed with a chemiluminescence reagent (ECL clarity, BioRad, Hercules, CA) and imaged by ChemiDoc MP (BioRad). Densitometric analysis was performed using Image Lab software (BioRad) on scanned nitrocellulose membranes obtained from a series of three independent blots each carried out using retinal samples from distinct experimental groups.

### Immunoprecipitation

Retinal extracts of AAV.iASPP or AAV.GFP injected eyes were immunoprecipitated with 2 μg of phosphoserine or control IgG antibodies (Millipore) following Catch and Release version 2.0 kit procedures (Millipore) and processed for Western blot analysis. Briefly, retinal extracts (400 μg) were incubated in continuous rotation for 3 hrs at 4°C in 500 μl of affinity beads carrying rabbit polyclonal anti-phosphoserine IgG (2 μg, Millipore). The beads were washed three times with wash buffer (Millipore), and the bound proteins were eluted by treating the beads twice with 70 μl of elution buffer. Detection and identification of immunoprecipitated proteins were performed by Western blots analysis as described above.

### Quantification of RGC survival

Rats were euthanized at one or two weeks post-axotomy by transcardial perfusion with 4% PFA and both the left (optic nerve lesion) and right (intact control) retinas were dissected and fixed for an additional 15 min. Brn3a immunodetection on whole-mounted retinas was performed as described [27]. Briefly, whole mounted retinas were permeabilized in PBS containing 0.5% Triton X-100 (Fisher, Waltham, MA) by freezing them at −80°C for 15 min, rinsed and incubated overnight at 4°C with goat-anti-Brn3a (0.27 μg/ml, Santa Cruz Biotechnologies, C-20) in blocking buffer (PBS, 2% normal donkey serum, 2% Triton X-100). Retinas were washed and incubated for 2 hrs at room temperature with Alexa Fluor donkey anti-goat IgG (1 μg/ml, Jackson ImmunoResearch Laboratories Inc.). Retinas were then rinsed, mounted vitreal side up, and covered with anti-fade solution (SlowFade, Molecular Probes, Eugene, OR). Brn3a-labeled neurons were counted within three square areas at distances of 1, 2 and 3 mm from the rat optic disc in each of the four retinal quadrants for a total of twelve retinal areas. Fluorescent staining was examined with a Zeiss Axioskop 2 Plus microscope (Carl Zeiss Canada, Kirkland, QC). Images were captured with a CCD video camera (Retiga, Qimaging, Burnaby, BC) and analyzed with Northern Eclipse software (Empix Imaging, Mississauga, ON).

### Statistical analyses

Data analysis and statistics were performed using the GraphPad Instat software (GraphPad Software Inc., San Diego, CA) by a one-way analysis of variance (ANOVA) followed by the Bonferroni *post hoc* test or Student's *t* test.

## Results

### iASPP is abundantly expressed by injured RGCs but its activity decreases after axonal damage

**Figure 1:**
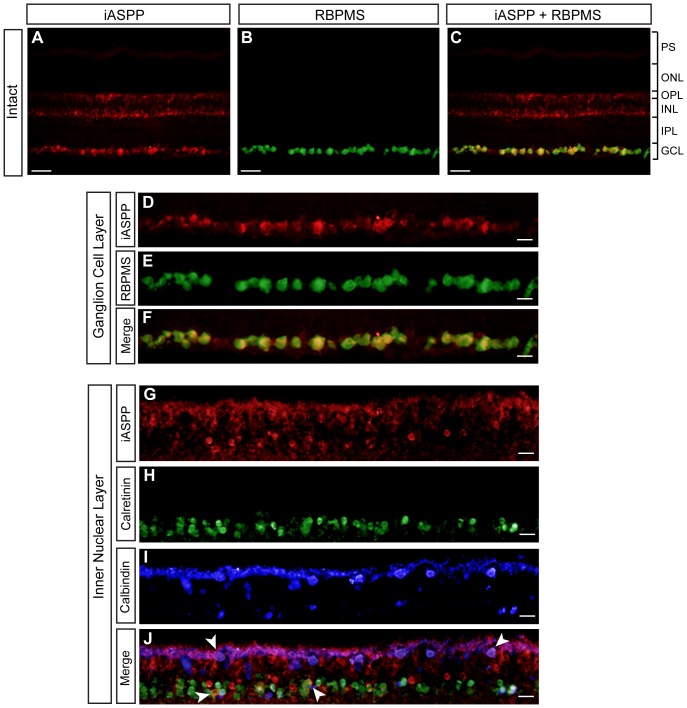
Adult RGCs express iASPP. Endogenous retinal iASPP was detected by immunofluorescence in the ganglion cell layer (GCL) and inner nuclear layer (INL) (A, C, D, and G). iASPP staining in RGCs was confirmed using the RGC-specific marker RBPMS (D–F). iASPP was also detected in amacrine and horizontal cells, visualized with calretinin (H,J, arrows) and calbindin (I,J, arrows), respectively. Scale bars: (A–C)  = 50 μm; (D–J)  = 20 μm. PS: Photoreceptor Segments; ONL: Outer Nuclear Layer; OPL: Outer Plexiform Layer; INL: Inner Nuclear Layer; IPL: Inner Plexiform Layer; GCL: Ganglion Cell Layer.

To characterize the role of the p53 inhibitor iASPP in RGC death, we first determined its cellular localization in the adult rat retina. Retinal immunohistochemistry showed expression of endogenous iASPP in the ganglion cell layer (GCL) and inner nuclear layer (INL) (Fig. 1A). As displaced amacrine cells account for ∼40–50% of the total number of neurons in the rat GCL [28], [29], we performed co-localization studies using antibodies against iASPP and ‘RNA binding protein with multiple splicing’ (RBPMS), a selective RGC marker [26], [30]. All RBPMS-positive neurons were immunoreactive for iASPP (Fig. 1B–F), indicating that adult RGCs are endowed with high levels of constitutive iASPP protein. In the INL, iASPP immunolabeling co-localized with calretinin, a marker of amacrine cells, and calbindin, a horizontal cell-specific marker (Fig. 1G–J), indicating that these cells also express iASPP. There was no co-localization between iASPP and PKCα suggesting that iASPP is not expressed by rod bipolar cells (not shown).

**Figure 2:**
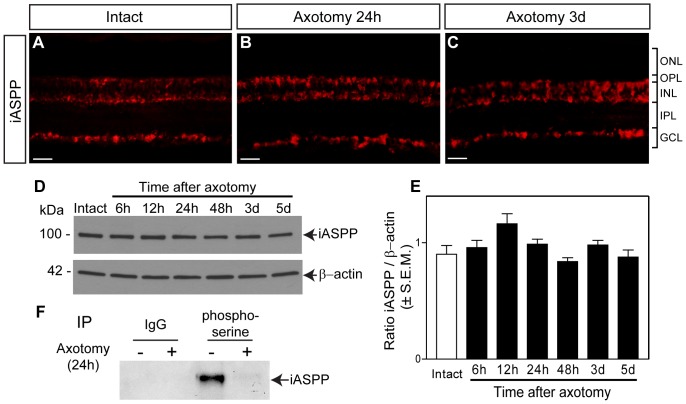
iASPP protein and phosphoserine levels after axotomy. Retinal iASPP expression and localization did not change at 24 hrs or 3 days after optic nerve injury compared to intact eyes (A–C). Scale bar: 10 μm. Analysis of protein homogenates from axotomized retinas collected at 6, 12, 24, 48 hrs, 3 and 5 days confirmed that iASPP levels were similar to those in intact, non-injured retinas. The lower panel represents the same blot as in the upper panel but probed with an antibody that recognizes β-actin used to confirm equal protein loading (D). Densitometric analysis of western blots, showing the ratio of iASPP protein relative to β-actin, confirmed that there is no significant change in protein levels after injury (E) (ANOVA, p>0.05). Phosphoserine immunoprecipitation (IP) of intact and axotomized retinas probed with iASPP antibody revealed a decrease in phosphoserine iASPP at 24 hrs after axotomy. IP of retinal homogenates with an IgG antibody was included as control for non-specific interactions (F). ONL: Outer Nuclear Layer; OPL: Outer Plexiform Layer; INL: Inner Nuclear Layer; IPL: Inner Plexiform Layer; GCL: Ganglion Cell Layer.

After axotomy, RGCs initially survive then die abruptly [16], [31], [32] and pro-apoptotic signals can be detected as early as 12 hrs after injury [33]. We did not detect changes in the levels or cellular localization of iASPP at 24 hrs or 3 days after axotomy (Fig. 2A–C), indicating that iASPP levels are similar in axotomized and non-injured retinas prior to the onset of RGC death. This finding was confirmed by western blot analysis of iASPP protein at 6 hrs, 12 hrs, 24 hrs, 48 hrs, 3 days and 5 days after axotomy (Fig. 2D, E). The phosphorylation of iASPP at serine residues increases its affinity towards p53 thus blocking the transcription of p53 pro-apoptotic target genes [34]. Therefore, we asked whether iASPP undergoes injury-induced changes in phosphorylation at serine residues. For this purpose, immunoprecipitation (IP) of endogenous phosphoserine proteins was performed on retinal lysates, and the eluates were probed with iASPP antibody to detect endogenous iASPP phosphorylated at serine residues. Phosphoserine IP showed enrichment of retinal iASPP in the intact eye, whereas no co-precipitation of iASPP was observed in axotomized retinas (Fig. 2F). Retinal lysates subjected to IP with an IgG antibody, to control for non-specific interactions, did not show detectable bands (Fig. 2F). Our finding suggests that optic nerve injury reduces iASPP phosphorylation at serine residues, which might compromise its ability to inhibit p53-mediated apoptosis.

### Retinal iASPP knockdown exacerbates RGC loss after axonal damage

**Figure 3:**
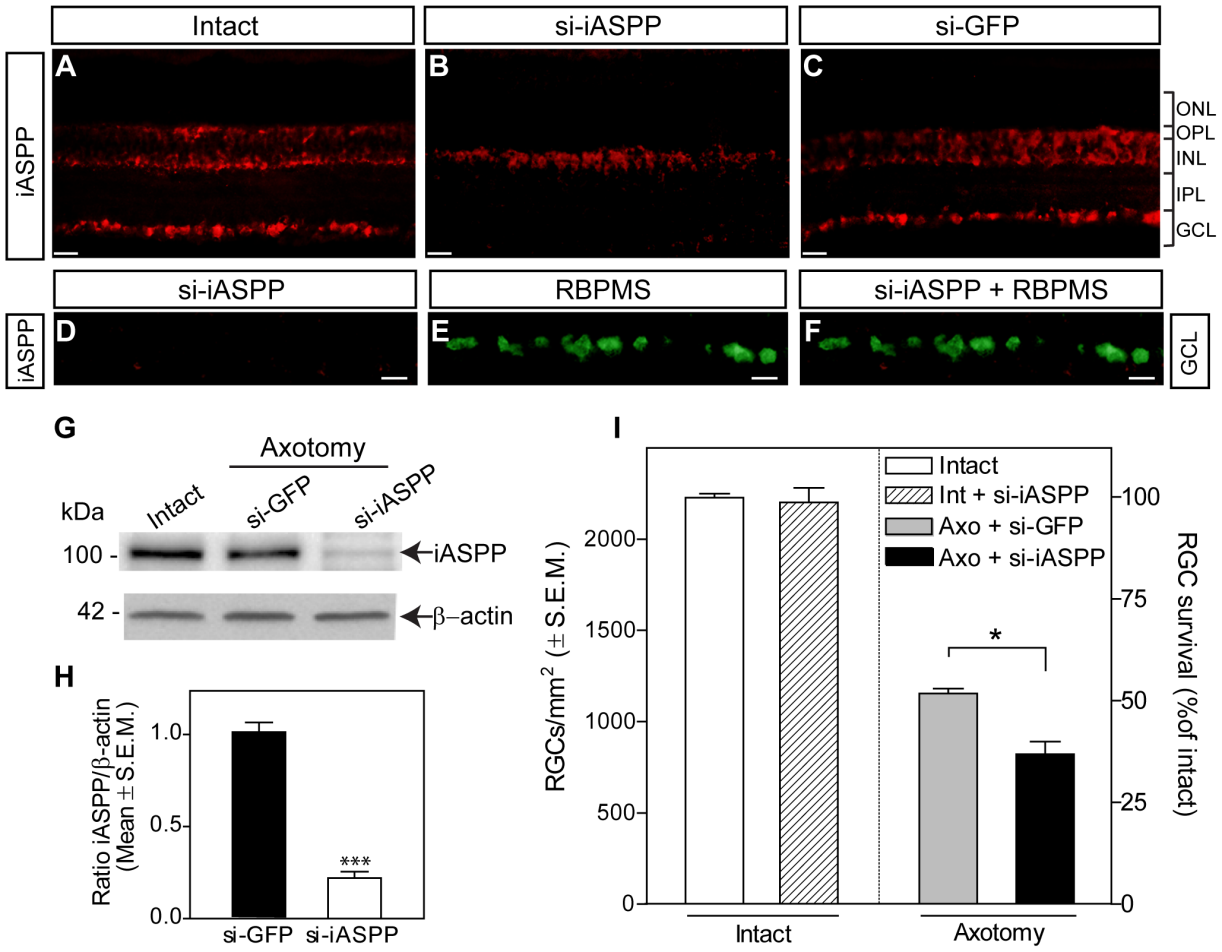
Selective siRNA knockdown of iASPP exacerbates axotomy-induced RGC death. A significant reduction of iASPP in the GCL was observed by immunohistochemistry of axotomized retinas at 24 hrs after intravitreal delivery of siRNA against iASPP (si-iASPP) compared to intact retinas, while control siRNA against GFP (siGFP) had no effect (A–C). RBPMS labeling confirmed that siRNA-mediated knockdown of iASPP occurred in RGCs (D–F). Scale bars: (A–C)  = 50 μm; (D–F)  = 15 μm. Western blot analysis confirmed that intravitreal delivery of si-iASPP led to marked reduction of retinal iASPP protein at 24 hrs after delivery, while siGFP had no effect (G, H; Student's T-test, *** = p<0.001). Quantitative analysis of RGC survival at one week after axotomy following intraocular injection of si-iASPP (black), or control siGFP (grey) (n = 4/group, ANOVA, * = p<0.05). The density of RGCs in intact, uninjured Sprague-Dawley rat retinas with si-iASPP intravitreal injection (hatched bar) or without (open bar) are shown as reference. Data are expressed as the mean ± S.E.M. ONL: Outer Nuclear Layer; OPL: Outer Plexiform Layer; INL: Inner Nuclear Layer; IPL: Inner Plexiform Layer; GCL: Ganglion Cell Layer.

To elucidate the role of iASPP in retinal neuron death, we first undertook a loss-of-function approach based on siRNA-mediated iASPP knockdown. We previously demonstrated that a single intravitreal injection of siRNA reaches the entire retina and rapidly downregulates target mRNAs in RGCs [18], [35]. Analysis of axotomized retinas treated with siRNA against iASPP (si-iASPP) at the time of axotomy revealed effective knockdown of iASPP protein in the GCL as early as 24 hrs after administration (Fig. 3A, B). Treatment with si-iASPP led to depletion of iASPP from RGCs, visualized with RBPMS (Fig. 3 D–F), whereas a control siRNA against GFP (si-GFP) had no effect (Fig. 3C). Similarly, western blot analysis confirmed robust knockdown of iASPP following si-iASPP administration in injured retinas (Fig. 3 G, H). Next, we asked whether iASPP depletion had an effect on axotomy-induced RGC loss. Quantitative analysis of Brn3a-labeled RGCs demonstrated that siRNA-mediated iASPP downregulation resulted in significantly greater RGC death (63%: 821±68 RGCs/mm^2^, n = 4) compared to siGFP-treated eyes (48%: 1154±27 RGCs/mm^2^, n = 4) at one week post-axotomy (Fig. 3I). Injection of si-iASPP into intact eyes did not change the density of RGCs compared to non-injured, non-treated controls ruling out the possibility that si-iASPP can induce RGC death by itself (Fig. 3I). These results indicate that loss of iASPP exacerbates RGC death following optic nerve injury.

### AAV-mediated iASPP overexpression selectively increases iASPP activity in RGCs

**Figure 4:**
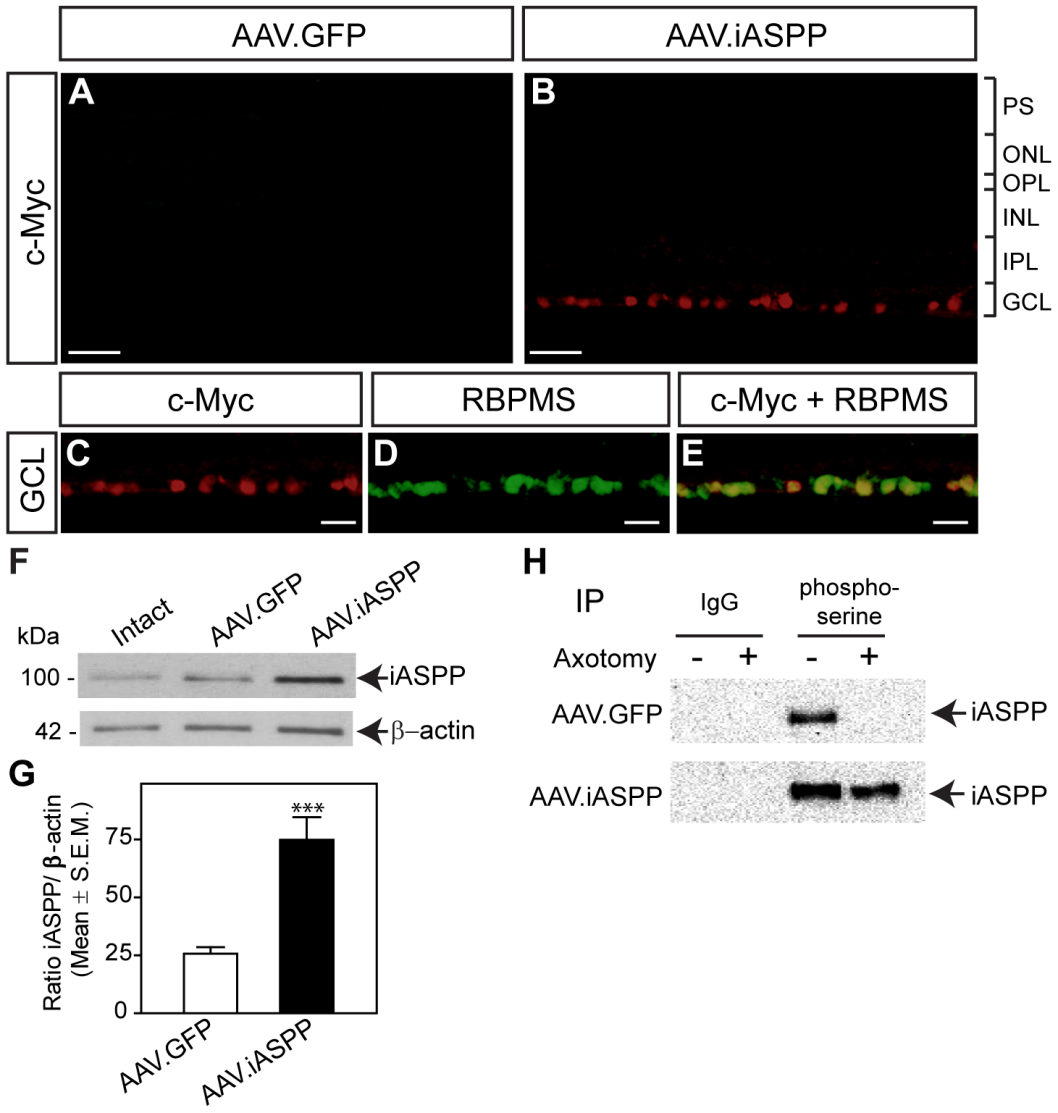
Targeted overexpression of iASPP in RGCs increases iASPP phosphoserine levels post-axotomy. AAV-mediated iASPP expression was distinguished from endogenous iASPP with an antibody against the c-myc tag encoded only in iASPP transgenes. Robust c-myc labeling was observed in the GCL of retinas that received AAV.iASPP, but not in control eyes injected with AAV.GFP (A, B). Selective expression of AAV-mediated iASPP in RGCs was confirmed using the RGC marker RBPMS (C–E). Scale bars: (A–B)  = 50 μm; (C–E)  = 15 μm. Immunoblotting and densitometric analyses confirmed that intravitreal delivery of AAV.iASPP led to significant overexpression of iASPP protein while AAV.GFP had no effect (F, G; Student's T-test, *** = p<0.001). Phosphoserine immunoprecipitation of retinas probed with an iASPP antibody reveals abundant iASPP phosphoserine levels in axotomized retinas (24 hrs) treated with AAV.iASPP but not with control AAV.GFP (H). PS: Photoreceptor Segments; ONL: Outer Nuclear Layer; OPL: Outer Plexiform Layer; INL: Inner Nuclear Layer; IPL: Inner Plexiform Layer; GCL: Ganglion Cell Layer.

AAV serotype 2 vectors were administered by intraocular injection to examine iASPP transgene expression in retinal cells *in vivo*. Retinas were examined two to four weeks following administration of AAV, the time required for optimal transgene expression using this vector [17], [19]. To distinguish AAV-mediated iASPP from endogenous iASPP, we used an antibody against the c-myc tag present only in the iASPP transgene. Robust c-myc staining was observed in a large number of cells in the GCL of retinas treated with AAV.iASPP, but not in control eyes injected with AAV.GFP (Fig. 4A, B). Co-localization of c-myc with RBMPS confirmed that AAV-transduced iASPP was expressed by RGCs (Fig. 4C–E). AAV.iASPP transduction occurred across the entire retina as previously shown by us [36]. Quantification of double-labeled c-myc and RBMPS-positive cells demonstrated that ∼85% of RGCs produced virally-encoded iASPP, consistent with previous reports showing high RGC transduction rates following intraocular administration of AAV serotype 2 [17], [19]. Western blot analysis confirmed virally-mediated iASPP upregulation in injured retinas, while control AAV.GFP had no effect (Fig. 4 F, G). Next, we asked whether overexpression of iASPP increased its availability to undergo serine phosphorylation after axotomy. IP experiments demonstrated a significant enrichment of iASPP phosphorylated at serine residues in axotomized retinas treated with AAV.iASPP, while no detectable iASPP phosphoserine was observed in AAV.GFP-treated injured retinas (Fig. 4H). Our data demonstrate that AAV-mediated iASPP expression increases the amount of serine phosphorylated iASPP in axotomized retinas.

### AAV.iASPP protects RGCs from axotomy-induced death

**Figure 5:**
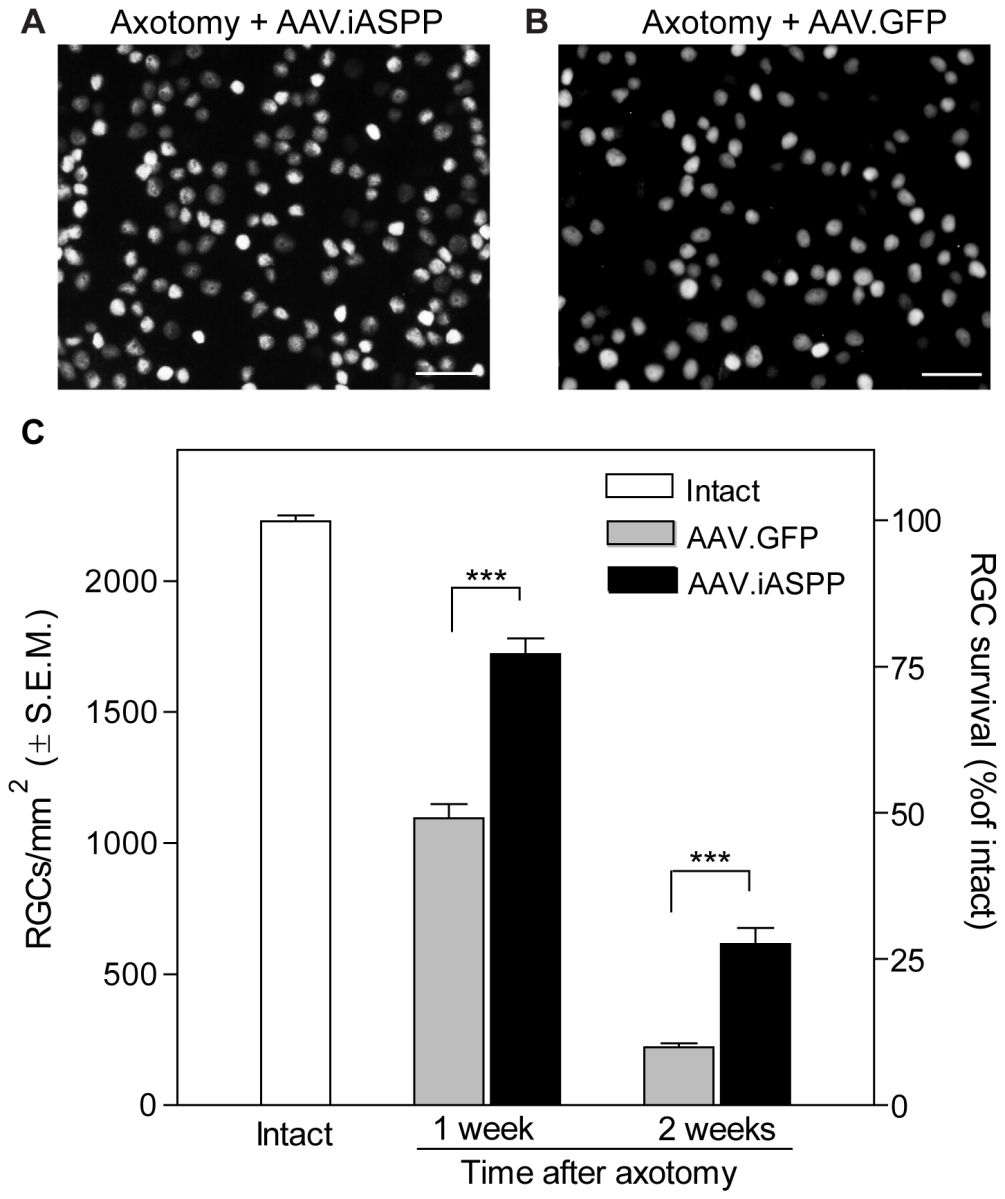
AAV-mediated iASPP overexpression increases RGC survival. Brn3a-labeled flat-mounted retinas from axotomized eyes demonstrate higher RGC densities following treatment with AAV.iASPP (A) than with AAV.GFP (B) at one week post-injury. Scale bars: 100 μm. Quantitative analysis of RGC survival following axotomy and intraocular injection of AAV.iASPP (black) or control AAV.GFP (grey) (ANOVA, *** = p<0.001) at one and two weeks post-injury (C). The density of RGCs in intact, uninjured Sprague-Dawley rat retinas is shown as reference (open bar). Data are expressed as the mean ± S.E.M.

The widespread expression of AAV-mediated iASPP in RGCs and its ability to activate iASPP *in vivo* prompted us to test its effect on RGC survival. For this purpose, intravitreal injections of AAV.iASPP or AAV.GFP were performed two weeks prior to axotomy and retinas were examined histologically at 7 and 14 days post-lesion to determine the density of surviving RGCs in all retinal quadrants. Flat-mounted retinas from eyes treated with AAV.iASPP showed higher densities of Brn3a-positive RGCs compared to AAV.GFP-treated control retinas (Fig. 5A, B). Quantitative analysis demonstrated that iASPP overexpression resulted in significant RGC survival (77%: 1720±61 RGCs/mm^2^, n = 5) with respect to eyes that received AAV.GFP (49%: 1094±55 RGCs/mm^2^, n = 5) at one week post-injury (Fig. 5 C). At two weeks after axotomy, only 9% of RGCs remained in eyes treated with AAV.GFP (221±14 RGCs/mm^2^, n = 5) whereas 27% of RGCs survived following AAV.iASPP treatment (615±31 RGCs/mm^2^, n = 4). Assessment of RGC numbers in all retinal areas analyzed (superior, temporal, inferior and nasal), indicated that the proportion of AAV.iASPP-mediated RGC survival compared to AAV.GFP was similar across retinal quadrants and eccentricities. These data indicate that AAV-mediated iASPP expression delays RGC death after injury and supports the conclusion that iASPP activity promotes RGC survival following axonal injury.

### iASPP downregulates p53 activity and the expression of pro-apoptotic targets PUMA and Fas/CD95

**Figure 6:**
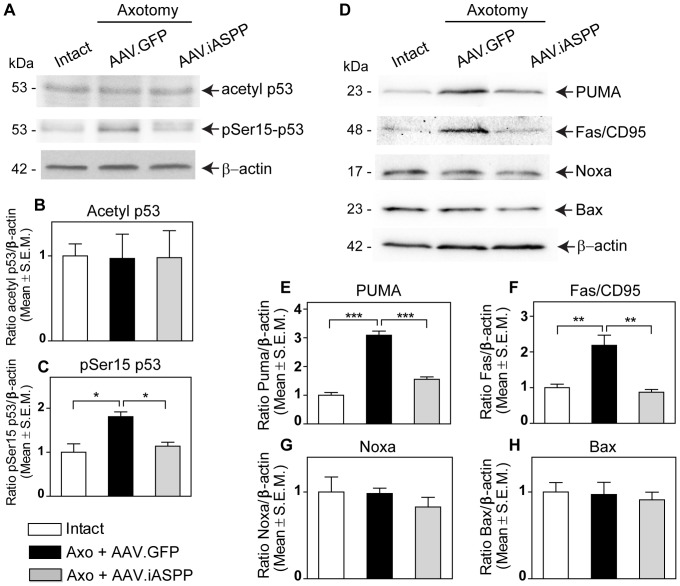
AAV.iASPP inhibits p53 activation and downregulates retinal PUMA and Fas/CD95 levels. Western blot analysis of axotomized retinal samples show that p53 phosphoserine15 (pSer15) levels are reduced in AAV.iASPP-treated retinas compared to control AAV.GFP at 24 hrs post-axotomy (A, C; ANOVA, * = p<0.05). Acetyl p53 levels remained unchanged (A, B; ANOVA, p>0.05). The p53 apoptotic targets PUMA and Fas/CD95 protein levels decrease in retinas treated with AAV.iASPP compared to AAV.GFP-treated control retinas (D, E, F; ANOVA, *** = p<0.005, ** = p<0.001), whereas Bax and Noxa remained unchanged (D, G, H; ANOVA, p>0.05).

To investigate the mechanisms by which iASPP overexpression might promote RGC survival, we first examined its effect on p53 post-translational modifications. Acetylation at p53 lysine residues 373 and 382 (Lys373, Lys382) by p300 occurs in the carboxyl-terminal region of p53 and has been correlated with its apoptotic function [37]–[39]. No changes in p53 acetylation at Lys373 and Lys382 were detected 24 hrs after axotomy in retinas treated with AAV.iASPP or control AAV.GFP (Fig. 6 A, B). In contrast, AAV.iASPP markedly inhibited the axotomy-induced increase in phosphoserine 15 (pSer15) p53 (Fig. 6 A, C), a key phosphorylation target during p53 activation [40], [41]. The reduction of pSer15 p53 in retinas overexpressing iASPP prompted us to assess the levels of the p53 pro-apoptotic targets PUMA, Fas/CD95, Bax and Noxa. Western blot analysis of retinal samples showed that AAV.iASPP markedly reduced PUMA and Fas/CD95 protein levels relative to control AAV.GFP (Fig. 6 D, E, F), while Bax and Noxa did not significantly change with any of the treatments (Fig. 6 D, G, H). We conclude that iASPP overexpression leads to downregulation of PUMA and Fas/CD95, suggesting that iASPP protects RGCs by inhibiting the ability of p53 to activate key pro-apoptotic targets.

## Discussion

The critical anti-apoptotic function of iASPP is underscored by its phylogenetic conservation, as it is the most evolutionarily conserved inhibitor of p53 [10]. Our study provides novel insight into the functional role of iASPP in neuronal survival, and allows us to draw the following conclusions. First, iASPP is abundantly expressed by adult RGCs, as well as a subset of amacrine and horizontal cells. Second, although total iASPP levels are not altered by optic nerve injury, phosphoserine iASPP levels, which serve as a readout of iASPP activity, were markedly reduced after axotomy. Third, selective knockdown of iASPP exacerbated RGC death while targeted iASPP overexpression increased phosphoserine iASPP levels and promoted RGC survival. Finally, we showed that AAV-mediated iASPP expression resulted in reduced p53 activity and rapid downregulation of pro-apoptotic targets PUMA and Fas/CD95. These data reveal a critical role for iASPP in the survival of CNS neurons following axonal injury.

We report constitutive expression of iASPP in RGCs, and in some amacrine and horizontal cells of the adult rat retina. RGCs express high levels of iASPP, however, a few faintly stained iASPP-positive cells in the GCL, which did not co-localize with RGC-specific markers, are most likely displaced amacrine cells. In the INL, there are some iASPP-positive cells that did not co-localize with either calretinin or calbindin suggesting that another type of amacrine cell, possibly glycinergic AII cells [42], also express iASPP. Although we previously showed that the expression of pro-apoptotic ASPP family members, ASPP1 and ASPP2, is restricted to the ganglion cell layer [18], another p53 inhibitor, MDM2, is also expressed by amacrine and horizontal cells in adult mice [43]. MDM4, which is structurally similar to MDM2, is also constitutively expressed in the adult retina [44], suggesting that complementary mechanisms are in place to ensure a tight regulation of p53 pro-apoptotic activity in retinal cells. Our finding that selective knockdown of iASPP by siRNA exacerbates RGC death after axonal injury is consistent with recent findings showing that downregulation of endogenous iASPP expression increases apoptosis in tumors of different origin including lung, breast, and prostate cancer as well as leukemia [45]–[48]. Indeed, the inhibition of iASPP has been proposed as a novel strategy for treating tumors affected by deregulation of p53 function. Of interest, our observation that AAV-mediated iASPP increases RGC survival following axotomy resembles the conferred resistance of iASPP-overexpressing cancer cells to chemotherapeutic drugs including placlitaxel [14], and cisplatin [1]. Thus, our complementary loss-of-function and gain-of-function experiments reveal a close parallel between the strong anti-apoptotic role of iASPP in cancer cells and that reported here for adult RGCs.

Our data show that retinal iASPP protein levels were not altered after optic nerve axotomy, similar to pro-apoptotic ASPP family members ASPP1 and ASPP2 [18]. Unlike cancer or stroke models in which the total level of ASPP has been shown to vary, this finding supports that, instead, iASPP phosphorylation is markedly reduced after axonal injury suggesting loss of iASPP activity in damaged neurons. The phosphorylation of iASPP at serine residues Ser84 and Ser113 sites was found to increase iASPP binding affinity to p53 [34]. PhosphoSitePlus [49], a curated protein phosphorylation site database identified by large scale Mass Spectrometry screening from various tissues and cell lines, reports the identification of at least 9 other serine phosphorylation residues in iASPP in addition to the Ser84 and Ser113 sites. Although not residue-specific, our phosphoserine immunoprecipitation assay demonstrates that iASPP is endogenously phosphorylated in the intact retina and that the level of iASPP serine phosphorylation is markedly reduced following optic nerve injury. Given that our immunoprecipitation assay was performed on whole retinal lysates, the observation that phospho-iASPP was not detected after axotomy suggests that iASPP phosphorylation is compromised in all the cells expressing this protein. However, we cannot rule out that iASPP phosphorylation in amacrine and horizontal cells does not change with axotomy but it is below detection levels. Notwithstanding, our data suggest that in the axotomized eye, the affinity of retinal iASPP towards p53 is reduced and, as such, may contribute to tilting the fate of injured neurons towards death. This hypothesis is strengthened by our finding that phosphoserine iASPP increases in AAV.iASPP-treated retinas, further supporting the conclusion that the affinity of iASPP towards p53 is greater in retinas overexpressing iASPP thereby blocking the apoptotic effect of p53 and enhancing cell survival.

To our knowledge, the only kinase known to phosphorylate iASPP is cyclin B1/CDK1 [34]. Of interest, the p53 downstream target and DNA damage-inducible protein Gadd45α has been shown to inhibit cyclin B1/CDK1 [50]. Gadd45α is upregulated in the ganglion cell layer following optic nerve transection and ocular hypertension [51]. Furthermore, Gadd45α modulates a positive feedback loop via p38, which phosphorylates p53 at its serine 15 site [52]. Therefore, it is possible that injury-induced Gadd45α upregulation inhibits cyclin B1/CDK1 thus preventing iASPP phosphorylation. We propose that, as an inhibitor of p53, iASPP overexpression might reduce p53-dependent transcription of Gadd45α, as evidenced by the decrease in pSer15-p53 levels reported here, allowing cyclin B1/CDK1 to phosphorylate iASPP.

We previously demonstrated that RGCs die in a p53-dependent manner following axonal damage and that although total p53 expression levels did not change after injury, critical post-translational modifications occurred *in vivo* [18]. We detected phosphorylation of p53 at serine 15, which has been shown to increase the ability of p53 to recruit CBP/p300 acetyltransferase [53]. However, we did not detect changes in acetylation of p53 on Lys 373 and Lys 382 soon after axotomy, which is consistent with a previous study in which neither acetylation at Lys373 nor p300 acetyltransferase levels were altered in RGCs 24 hrs after optic nerve crush [54]. Therefore, although acetylation at Lys373 and Lys382 are known to be involved in fine-tuning the p53 stress response [55], these modifications do not appear to play a critical role in early changes associated with RGC death. In agreement with this, the loss of p53 acetylation at its C terminus by CBP/p300 was not required for p53 transactivation in an acetylation-deficient missense mutant mouse model [55].

Along with an increased affinity towards p53, phosphorylated iASPP has been reported to reduce pro-apoptotic gene transcription [34]. Indeed, iASPP phosphorylation at Ser84 and Ser113 resulted in reduced transcriptional activity of p53 targets PUMA, Bax and PIG3 compared to wild-type ASPP in melanoma cells [34]. Similarly, we show that AAV-iASPP significantly increases the levels of phosphoserine iASPP, which coincided with reduced levels of PUMA and Fas/CD95, leading to neuronal survival. Of interest, siRNA-mediated knockdown of PUMA or Fas/CD95 resulted in substantial RGC protection after optic nerve injury [18]. Decreased PUMA and Fas/CD95 expression may rescue RGCs by affecting intrinsic and extrinsic apoptotic pathways, respectively. Active PUMA, a BH3-only Bcl-2 family member and critical mediator of p53-dependent apoptosis [56], may act indirectly on pro-apoptotic Bcl-2 family members by relieving the inhibition imposed by anti-apoptotic members [57], [58]. Fas/CD95, a death receptor that triggers apoptosis when bound by Fas ligand after recruiting the adapter protein FADD (Fas-associated death domain) and pro-caspase 8 [59], is weakly expressed in the intact rodent retina [60], [61]. The expression of Fas/CD95 markedly increases in the ganglion cell layer as well as microglia during glaucomatous damage [60], [62]. Furthermore, FADD is upregulated in RGCs subjected to ocular hypertension [62], and FasL-expressing microglia can induce apoptotic RGC death in a spontaneous mouse glaucoma model [63]. The reduction of Fas/CD95 levels reported here following iASPP.AAV administration may effectively decrease the activation of death receptor apoptotic pathways mediated by FasL, thereby increasing RGC survival. Overall, our data suggest that iASPP expression in RGCs is required as a molecular checkpoint to ensure that p53 activity is kept low and under tight control in healthy cells. Specifically, iASPP is likely to inhibit the ability of p53 to stimulate pro-apoptotic retinal targets, including PUMA and Fas/CD95, thus preventing or attenuating p53-dependent neuronal death.

In conclusion, we identify a novel role for the highly conserved p53 inhibitor iASPP in the survival of retinal neurons subjected to axonal injury. Our findings expand our current understanding of the role of the ASPP family of p53 regulators in neurodegeneration which could prove beneficial for the design of strategies aimed at curtailing neuronal loss in the injured CNS.
