# Correction: Inhibitor of Apoptosis-Stimulating Protein of p53 (iASPP) Is Required for Neuronal Survival after Axonal Injury

**DOI:** 10.1371/journal.pone.0343169

**Published:** 2026-02-18

**Authors:** Ariel M. Wilson, Vince A. Chiodo, Sanford L. Boye, Nicholas C. Brecha, William W. Hauswirth, Adriana Di Polo

After publication of this article [1], concerns were raised regarding Figs 1-3 and 6. Specifically:

- Figs 1A and C appear similar to Fig 2A and Fig 3A.
- Fig 2C appears similar to Fig 3C.
- The β-actin panel of Fig 3G appears similar to the β-actin panel of Fig 6A when flipped horizontally.
- When levels are adjusted to visualize background, there appears to be a vertical discontinuity between lanes 2 and 3 in the PUMA panel of Fig 6D.

The corresponding author, ADP, stated that in Figs 1A, 2A and 3A, the same representative photomicrograph of an intact/uninjured retina was intentionally used across multiple figs to illustrate baseline retinal morphology. They also stated that Fig 3C is incorrect and Fig 2C is correct. The Fig 3C immunohistochemistry panel has been replaced with the correct image from the original dataset in the updated Fig 3 in S1 File. Underlying data in support of Figs 1-3 are provided here in S2-S3 and S5-S6 Files.

The corresponding author stated that the β-actin blot in Fig 6A is incorrect, and the β-actin blot in Fig 3G is correct. The β-actin panel in Fig 6A has been replaced with the correct loading control derived from the original experiment in the updated Fig 6 provided here. The corresponding author also stated that they were unable to locate the original uncropped western blots for Fig 3G, and the specific PUMA western blot image originally used to assemble Fig 6D. They have therefore provided uncropped western blots from parallel experiments conducted at the same time as the original experiments and used in the original quantitative analyses for Figs 3G and 6D in S3-S4 Files. Fig 6D has been replaced with these alternative blots generated at the time of the initial experiments in the updated Fig 6 provided here. Underlying data in support of the updated Fig 6 are provided here in S4 and S6 Files.

The corresponding author stated that the remainder of the underlying data supporting [1] are available upon request, with the exception of the individual-level quantitative data for the intact iASPP siRNA-treated retinal ganglion cell counts in Fig 3I, a non-injured control group.

**Fig 6:**
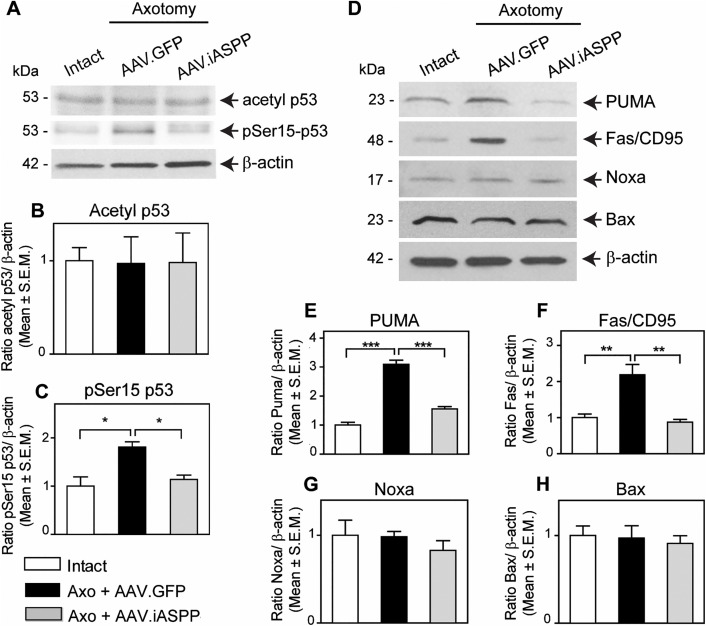
AAV.iASPP inhibits p53 activation and downregulates retinal PUMA and Fas/CD95 levels. Western blot analysis of axotomized retinal samples show that p53 phosphoserine15 (pSer15) levels are reduced in AAV.iASPP-treated retinas compared to control AAV.GFP at 24 hrs post-axotomy (A, C; ANOVA, * = p < 0.05). Acetyl p53 levels remained unchanged (A, B; ANOVA, p > 0.05). The p53 apoptotic targets PUMA and Fas/ CD95 protein levels decrease in retinas treated with AAV.iASPP compared to AAV.GFP-treated control retinas (D, E, F; ANOVA, *** = p < 0.005, ** = p < 0.001), whereas Bax and Noxa remained unchanged (D, G, H; ANOVA, p > 0.05).

## Supporting information

S1 File Updated version of Fig 3 with correct Fig 3C. (ZIP)

S2 File Underlying data in support of Figs 2B-E and a replicate representative image of an intact retina from the time of the original experiments in support of Fig 2A. (ZIP)

S3 File Underlying data in support of Figs 3H and I (intact, axotomy + siGFP, axotomy + si-iASPP only), underlying data in support of Figs 3B and D-F, replicate representative underlying western blots from the time of the original experiments in support of Fig 3G, the correct underlying image for the updated Fig 3C (S1 File), and a replicate representative image of an intact retina from the time of the original experiments in support of Fig 3A. (ZIP)

S4 File Underlying data in support of Figs 6B-C and E-H and the updated Fig 6A, and replicate representative underlying western blots from the time of the original experiments used in the updated Fig 6D. (ZIP)

S5 File Underlying data in support of Figs 1A-C, 2A and 3A. (ZIP)

S6 File Individual-level underlying data in support of Figs 2E, 3H-I (intact, axotomy + siGFP, axotomy + si-iASPP only), 6B-C, and 6E-H, including means SEMs and underlying values used to determine ratios. (ZIP)
