# Supplementary figures and images for: Correction: Inhibitor of Apoptosis-Stimulating Protein of p53 (iASPP) Is Required for Neuronal Survival after Axonal Injury
Source: PLoS One. 2026 Feb 18;21(2):e0343169. doi: 10.1371/journal.pone.0343169 (PMC12915942; doi:10.1371/journal.pone.0343169)

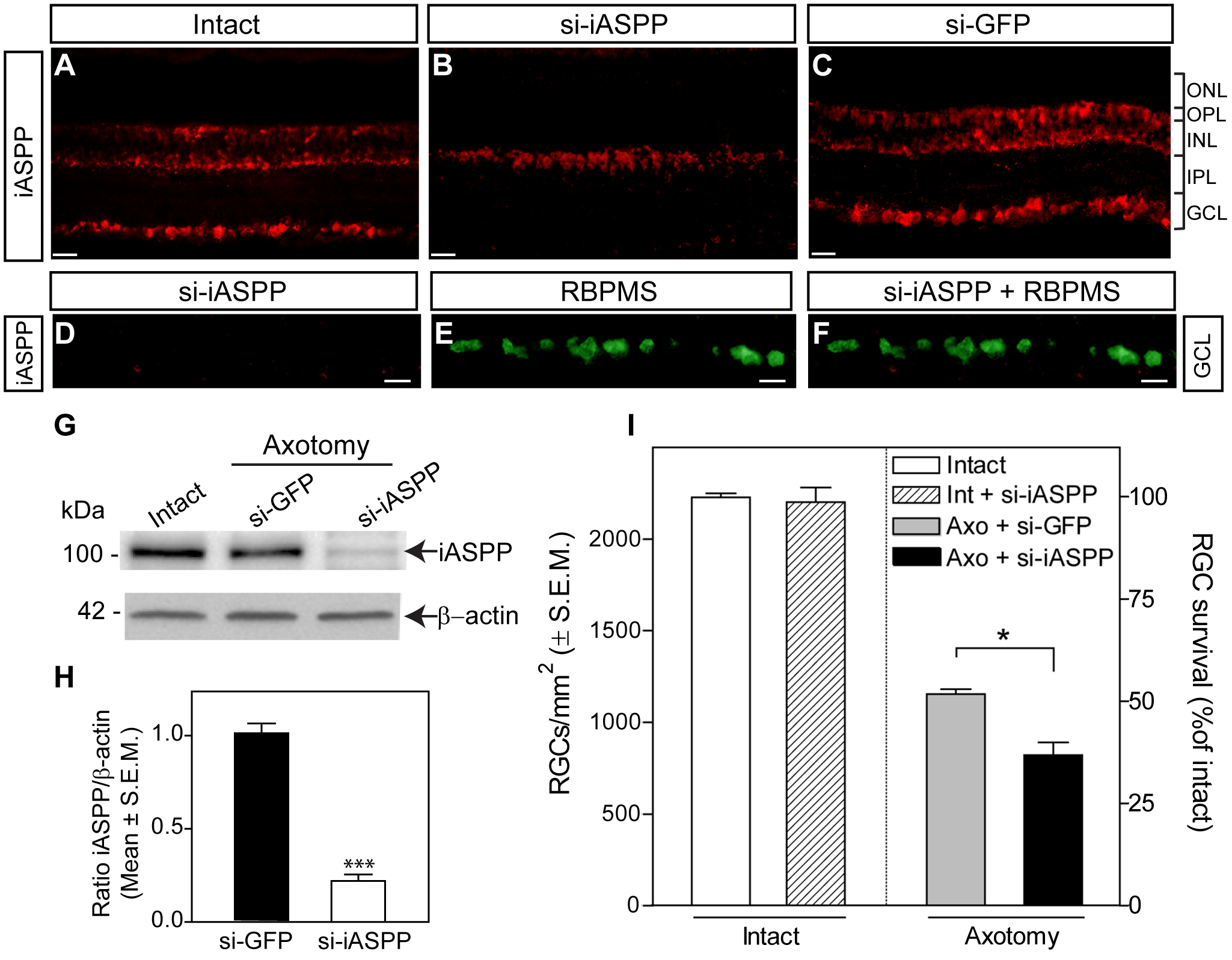

Supplement: S1 File — (ZIP) [file pone.0343169.s001.zip › S1 File/Figure 3 with original Fig3A.tif]

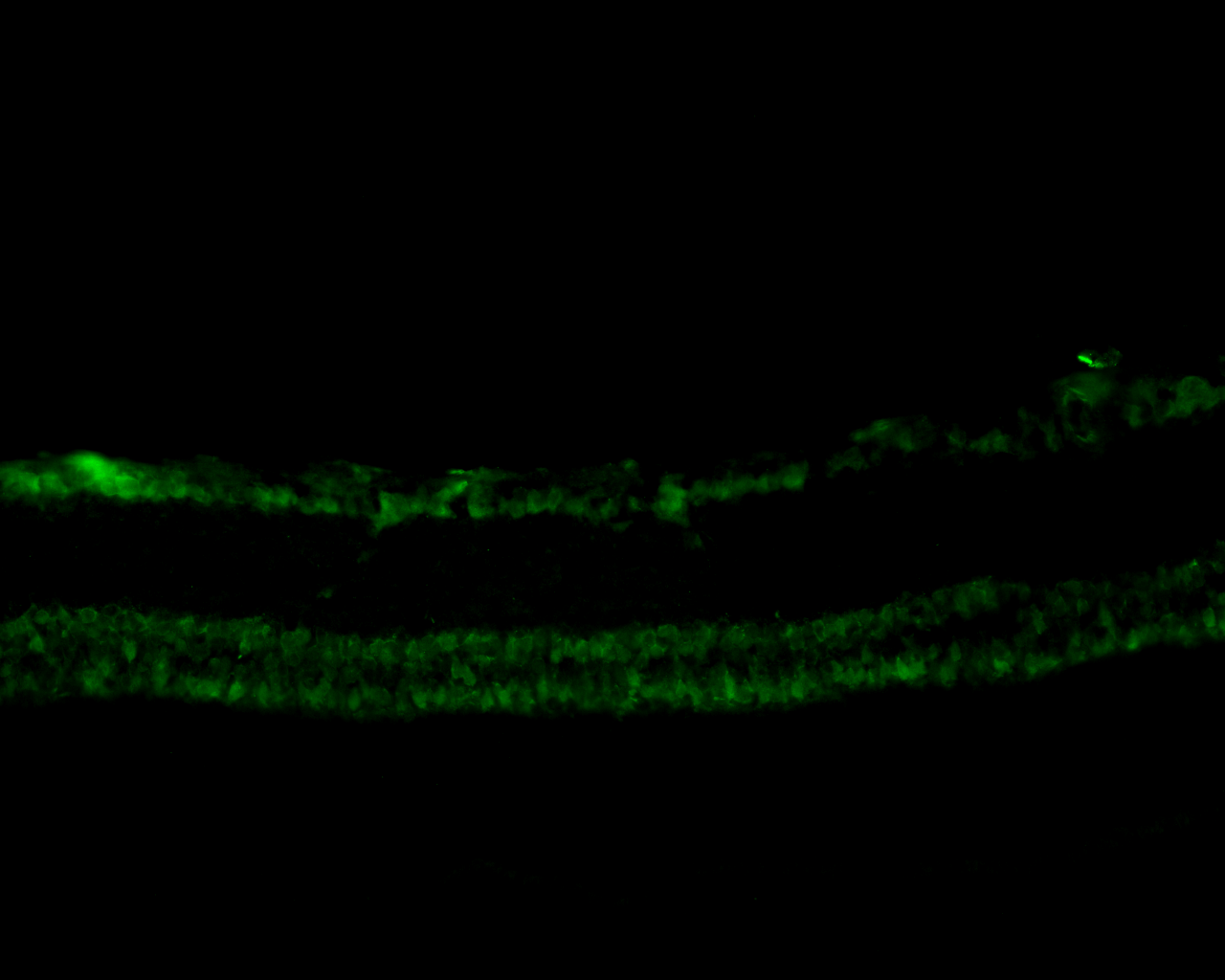

Supplement: S2 File — (ZIP) [file pone.0343169.s002.zip › S2 File/Fig2B_iASPP_axo24.tif]

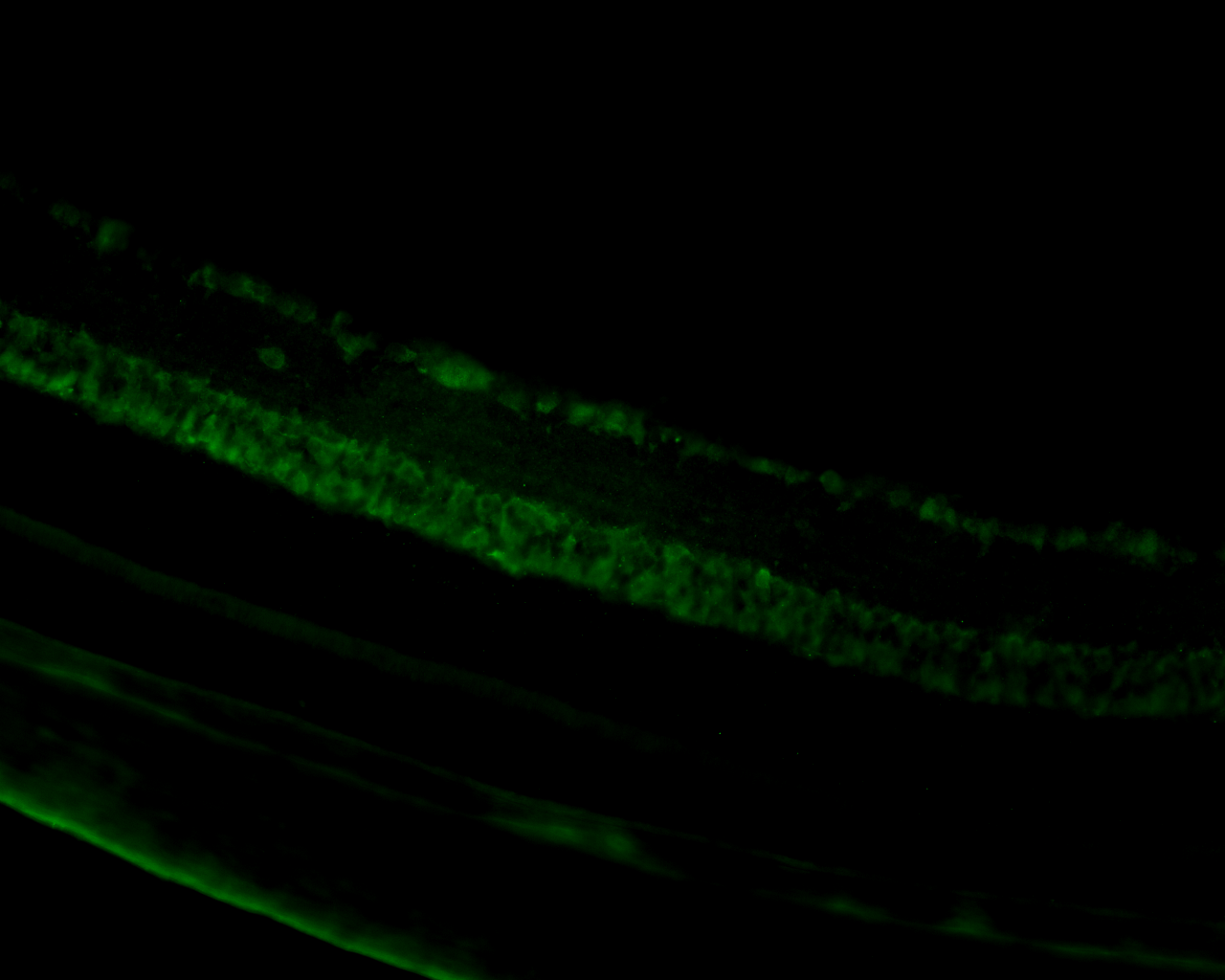

Supplement: S2 File — (ZIP) [file pone.0343169.s002.zip › S2 File/Fig2C_iASPP_axo3d.tif]

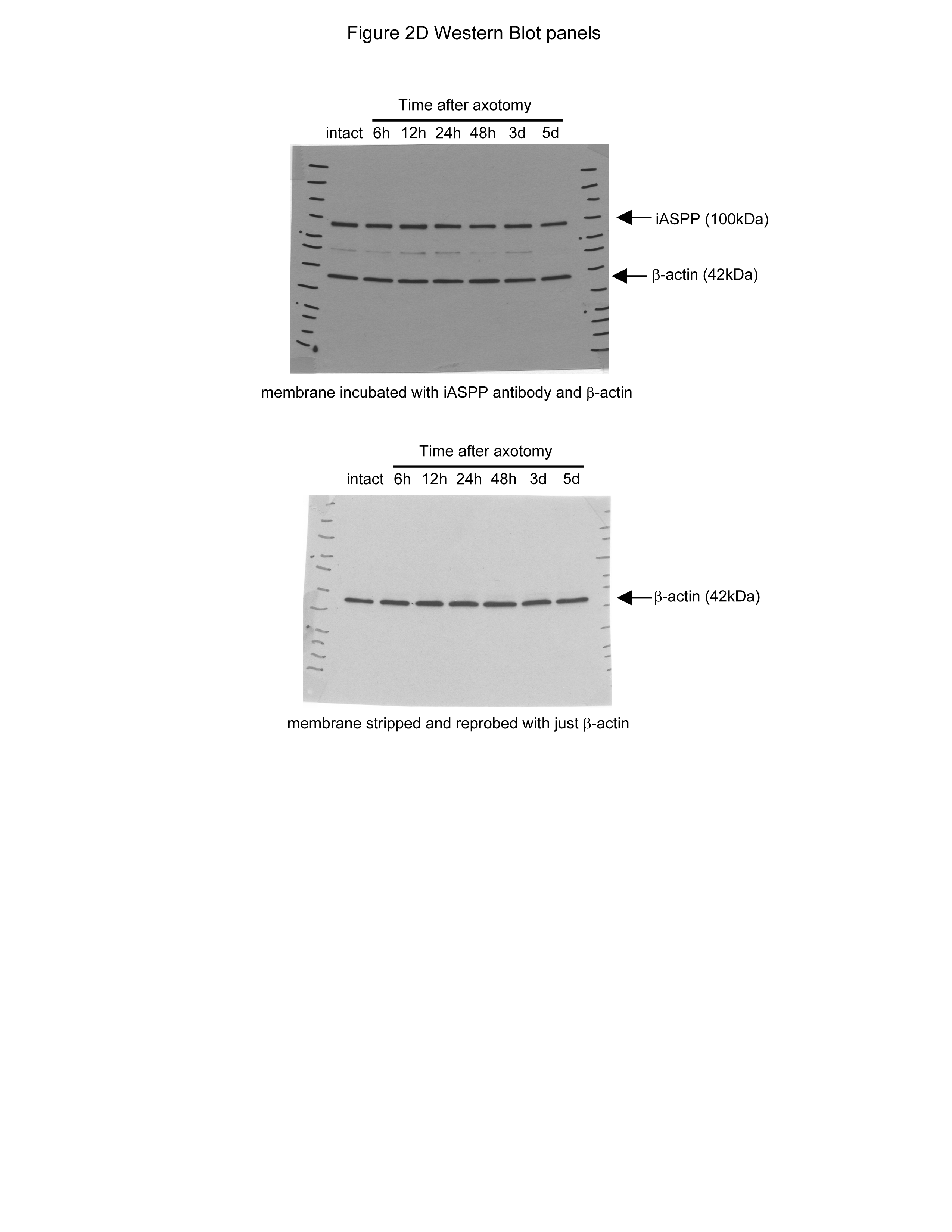

Supplement: S2 File — (ZIP) [file pone.0343169.s002.zip › S2 File/Fig2D_WesternBlots_axoiASPP.tif]

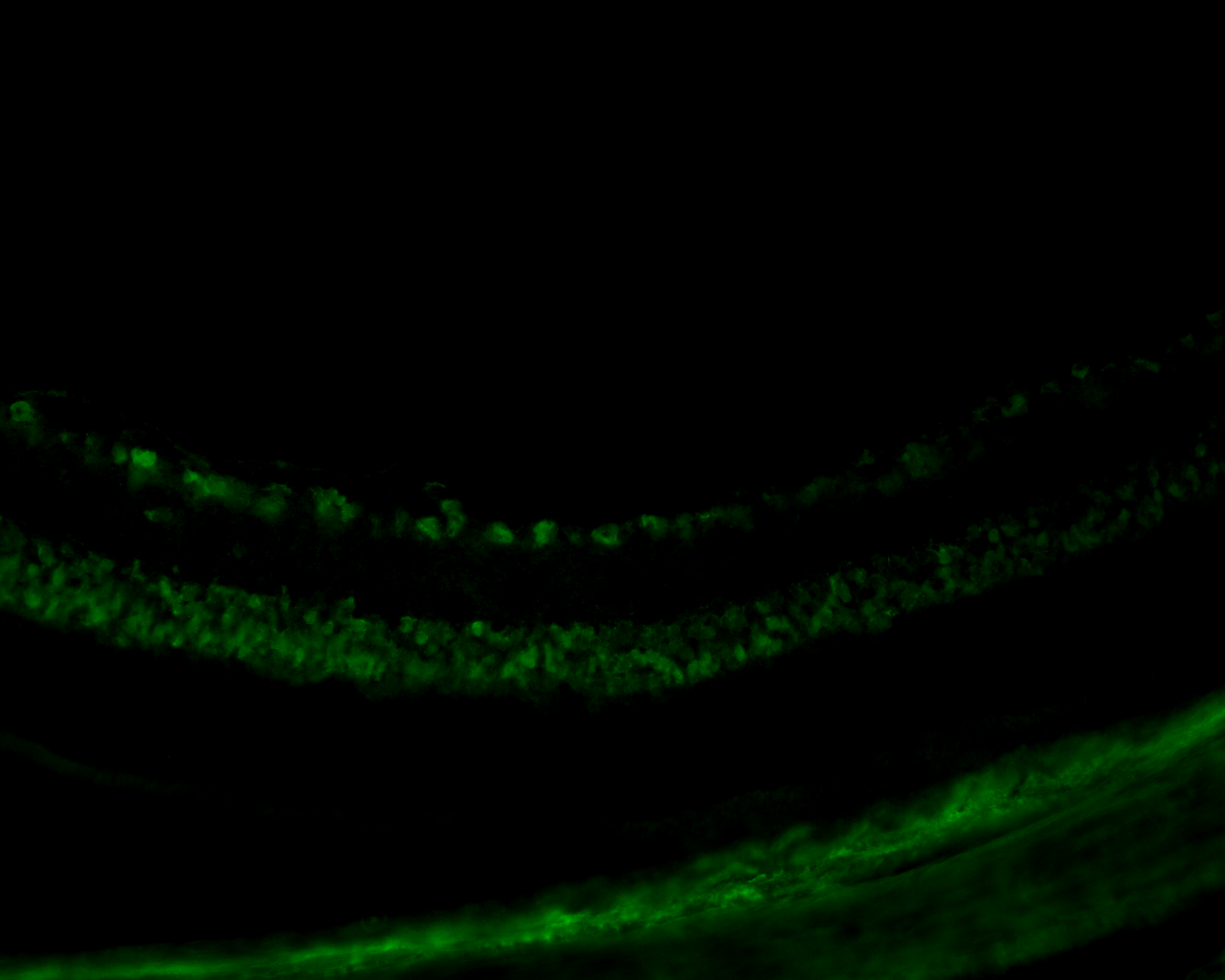

Supplement: S3 File — (ZIP) [file pone.0343169.s003.zip › S3 File/Fig3A_iASPP_intact_newpanel.tif]

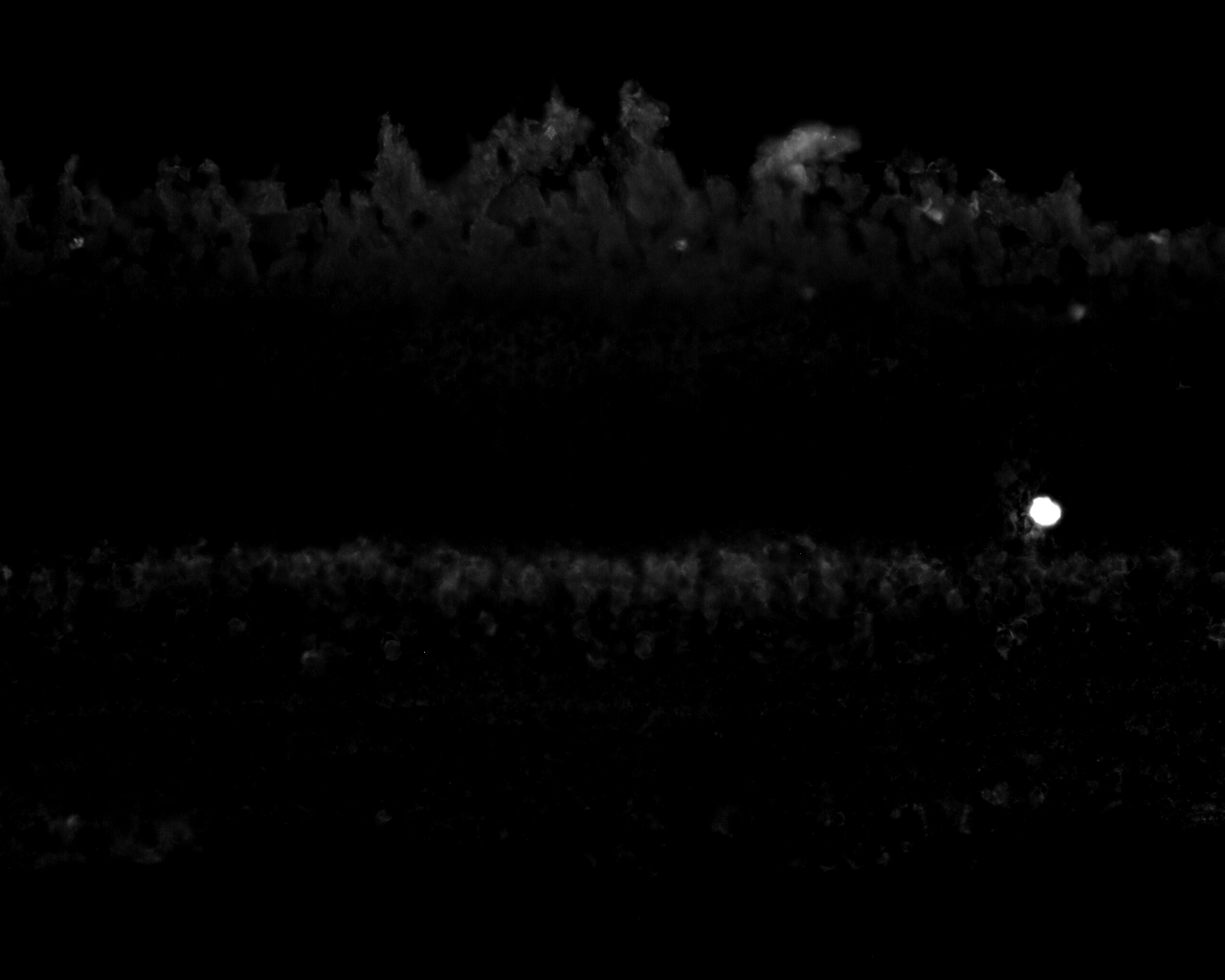

Supplement: S3 File — (ZIP) [file pone.0343169.s003.zip › S3 File/Fig3B_siiASPP.tif]

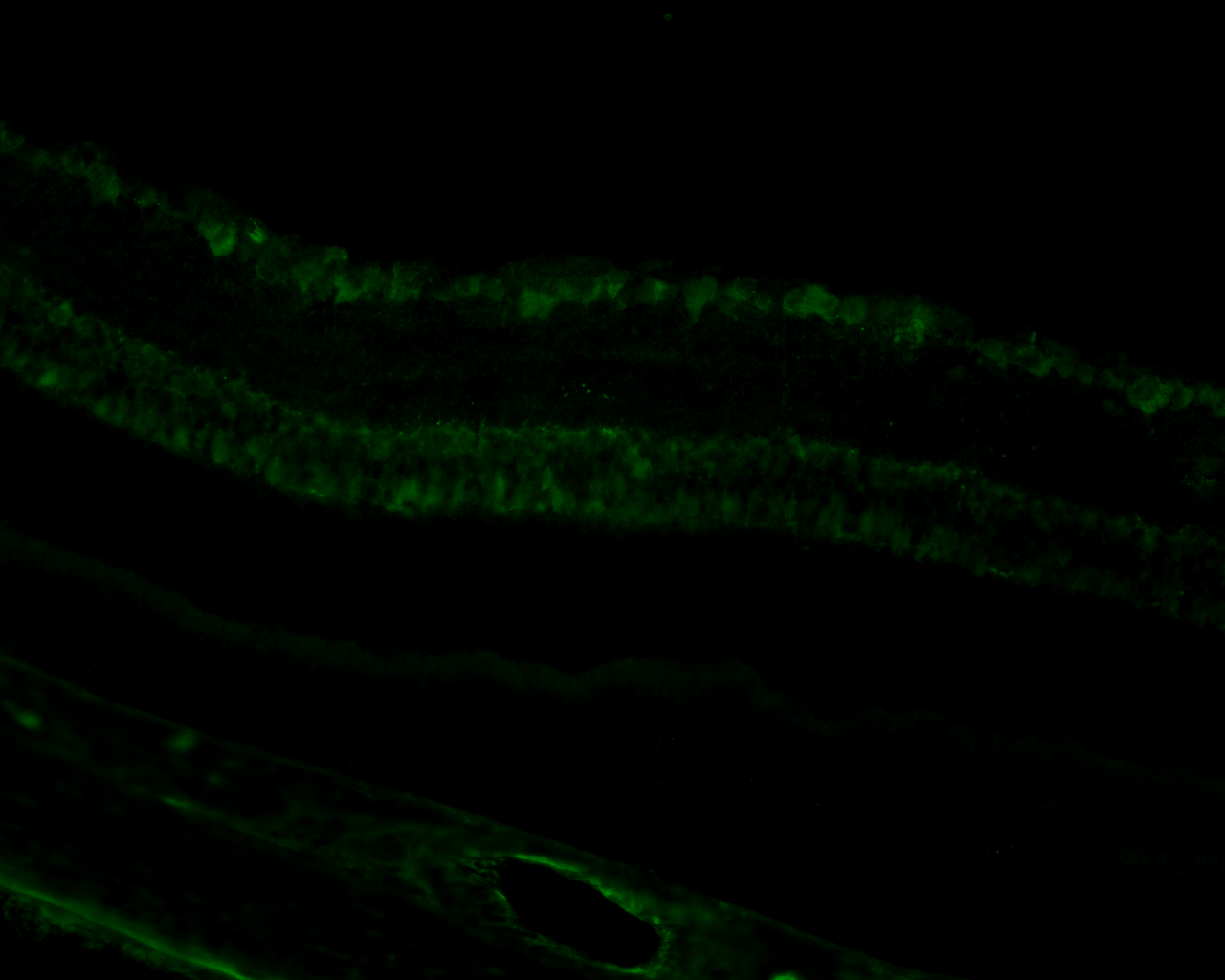

Supplement: S3 File — (ZIP) [file pone.0343169.s003.zip › S3 File/Fig3C_siGFP_newpanel.tif]

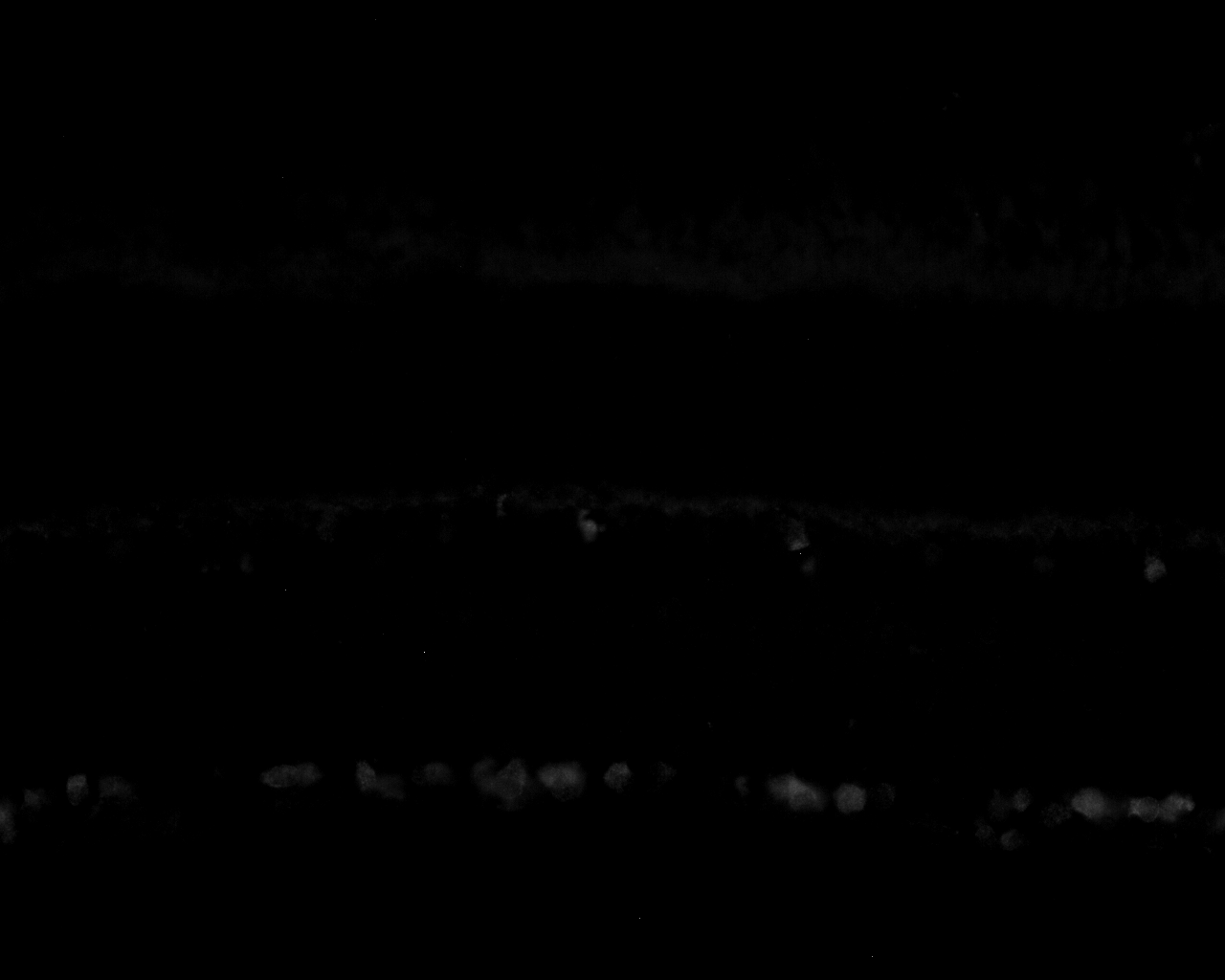

Supplement: S3 File — (ZIP) [file pone.0343169.s003.zip › S3 File/Fig3E_RBPMS.tif]

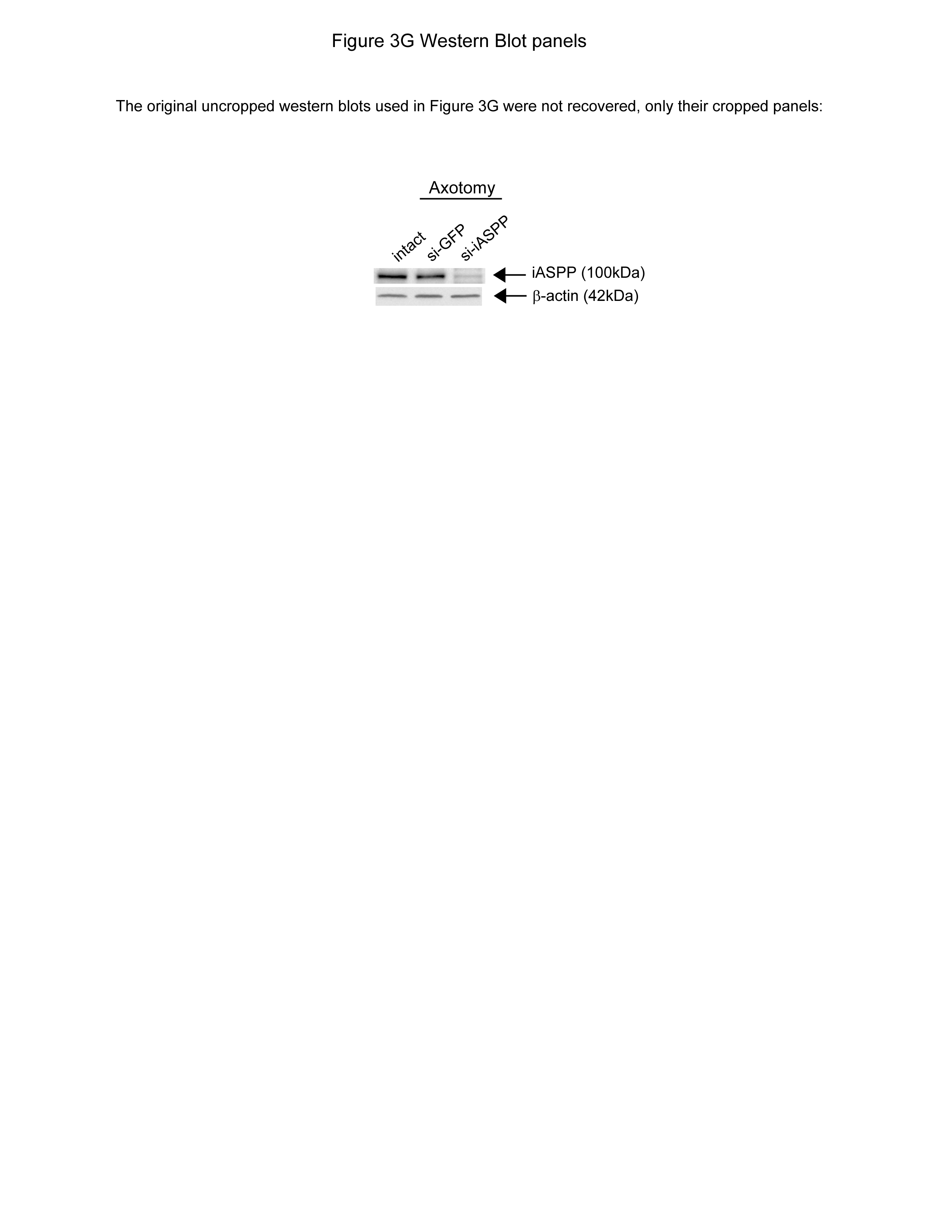

Supplement: S3 File — (ZIP) [file pone.0343169.s003.zip › S3 File/Fig3G_WesternBlots_siiASPP.tif]

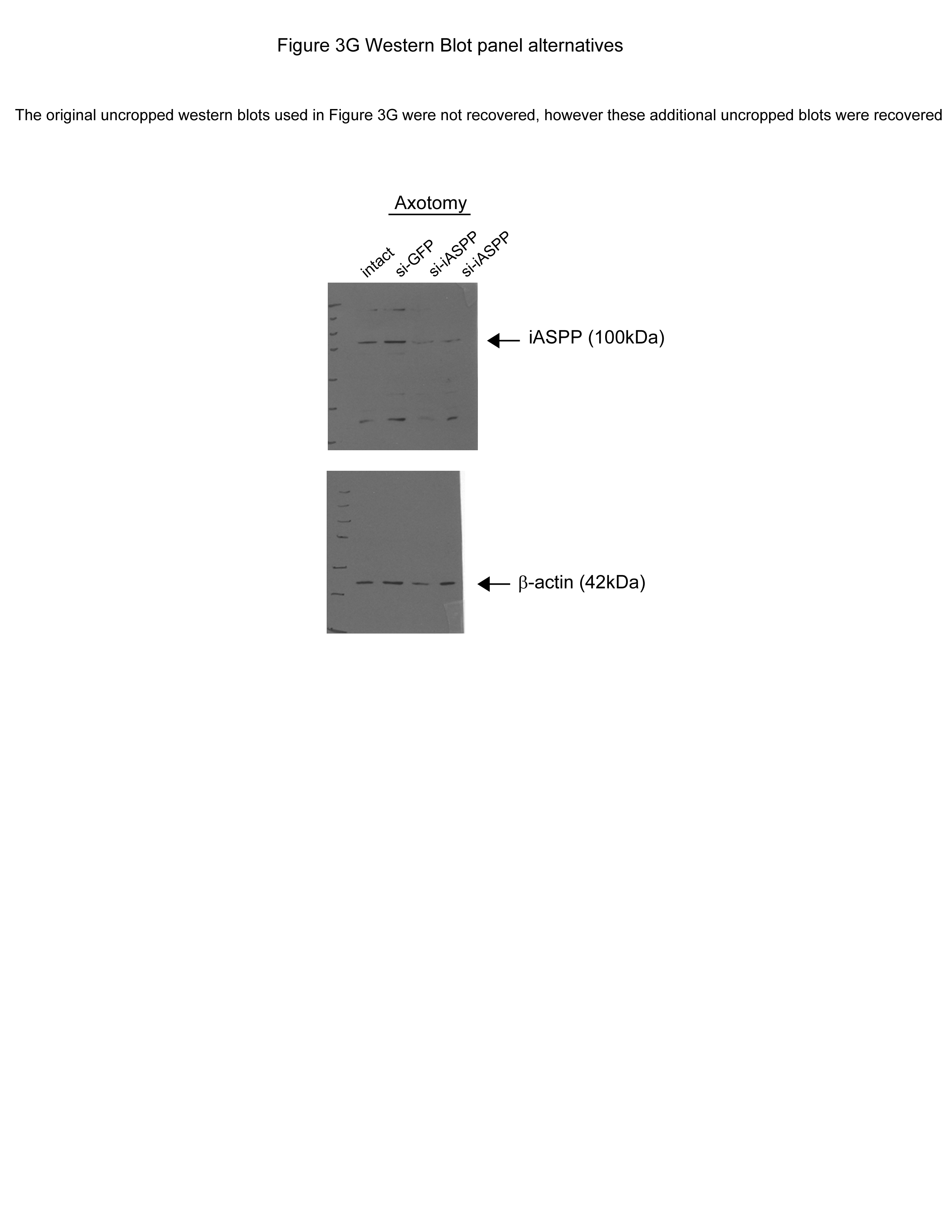

Supplement: S3 File — (ZIP) [file pone.0343169.s003.zip › S3 File/Fig3G_WesternBlots_siiASPP_alternative.tif]

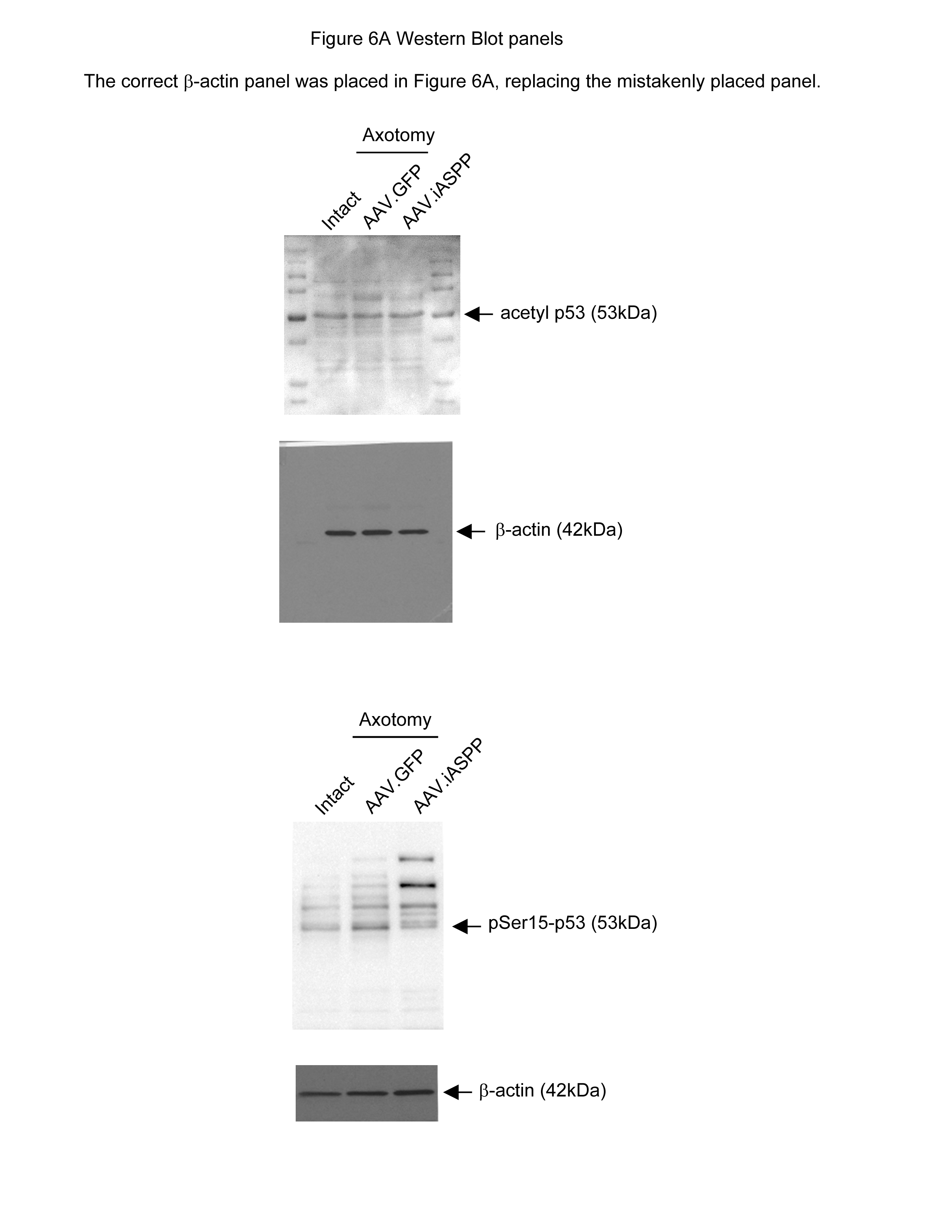

Supplement: S4 File — (ZIP) [file pone.0343169.s004.zip › S4 File/Fig6A_WesternBlots_acetylp53_pSer15p53.tif]

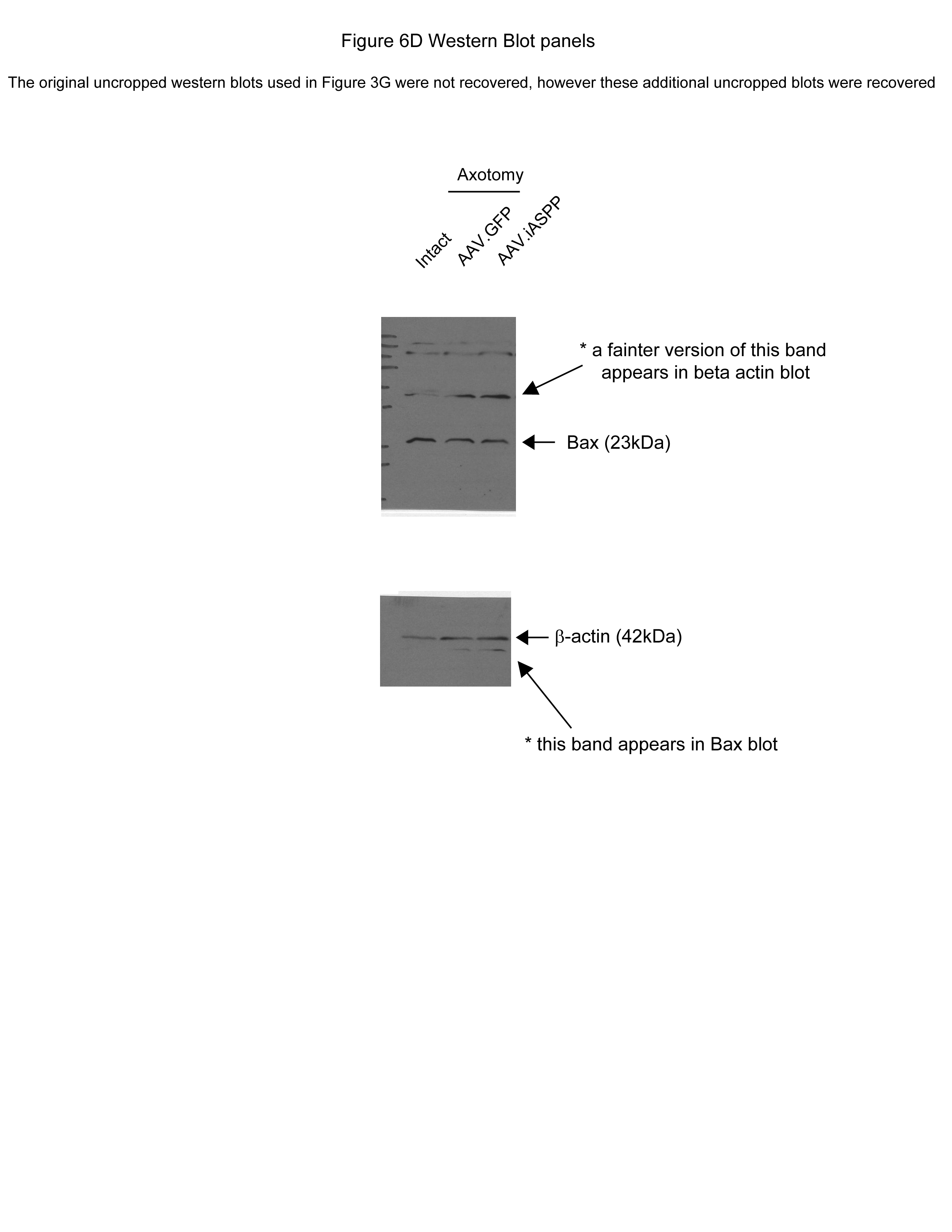

Supplement: S4 File — (ZIP) [file pone.0343169.s004.zip › S4 File/Fig6D_WesternBlots_Bax.tif]

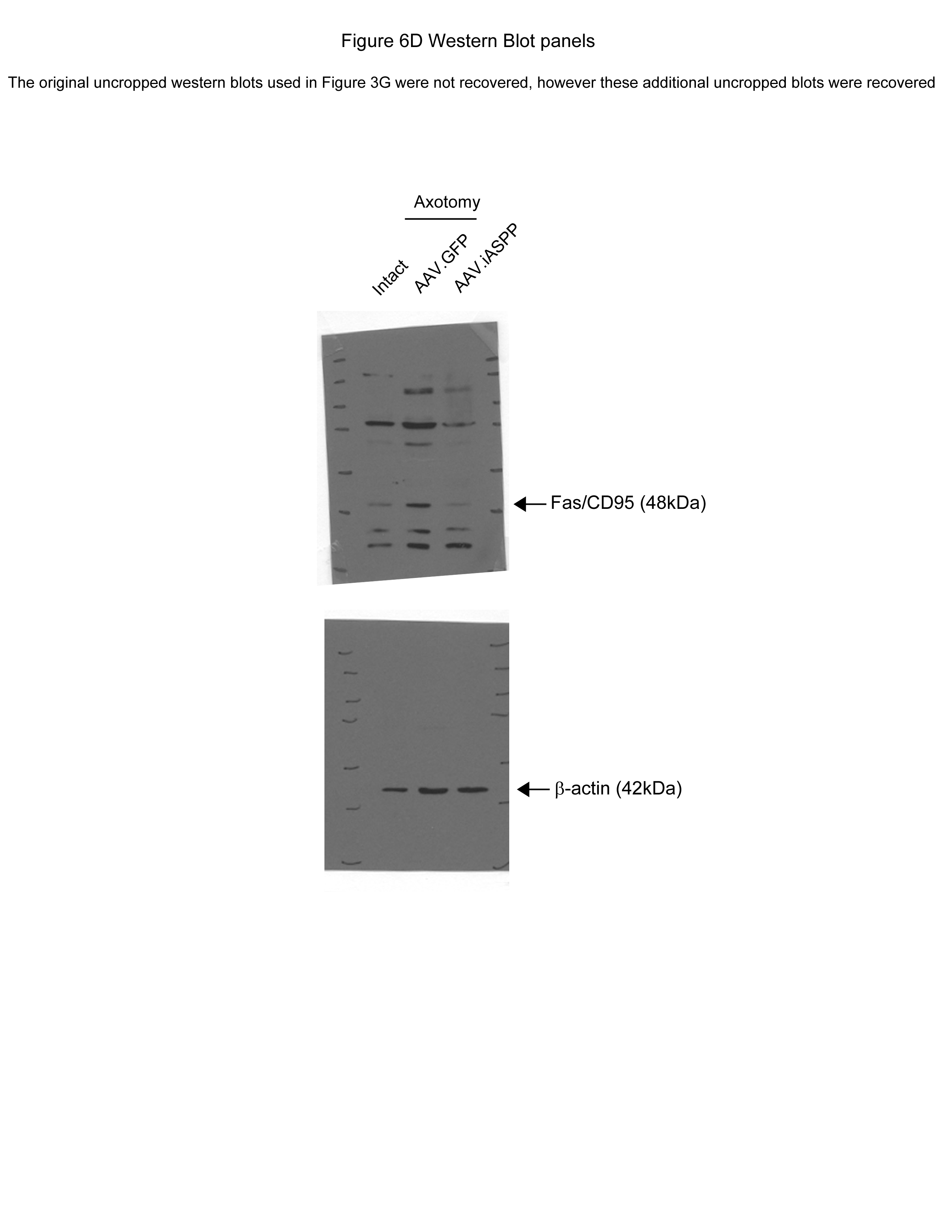

Supplement: S4 File — (ZIP) [file pone.0343169.s004.zip › S4 File/Fig6D_WesternBlots_FasCD95.tif]

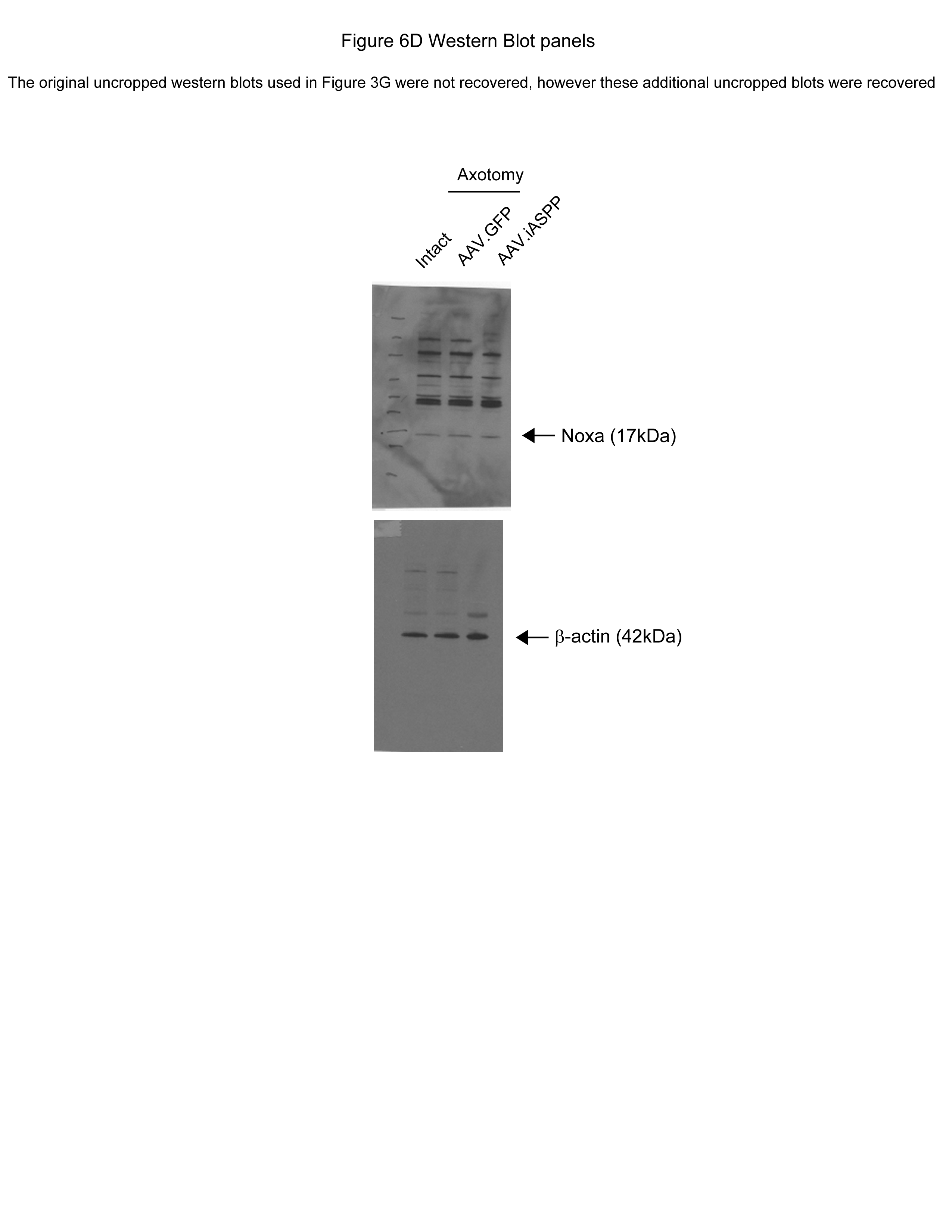

Supplement: S4 File — (ZIP) [file pone.0343169.s004.zip › S4 File/Fig6D_WesternBlots_Noxa.tif]

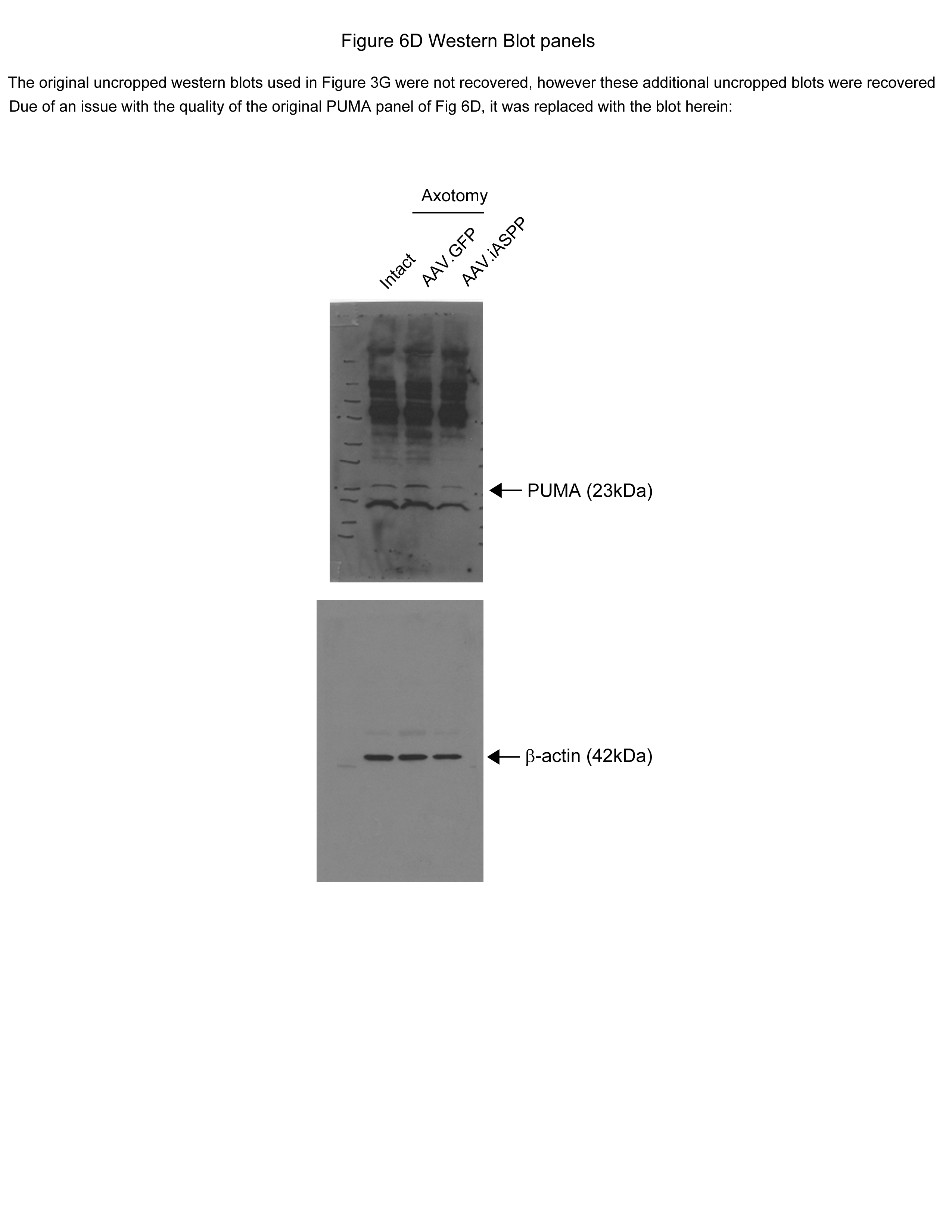

Supplement: S4 File — (ZIP) [file pone.0343169.s004.zip › S4 File/Fig6D_WesternBlots_PUMA.tif]

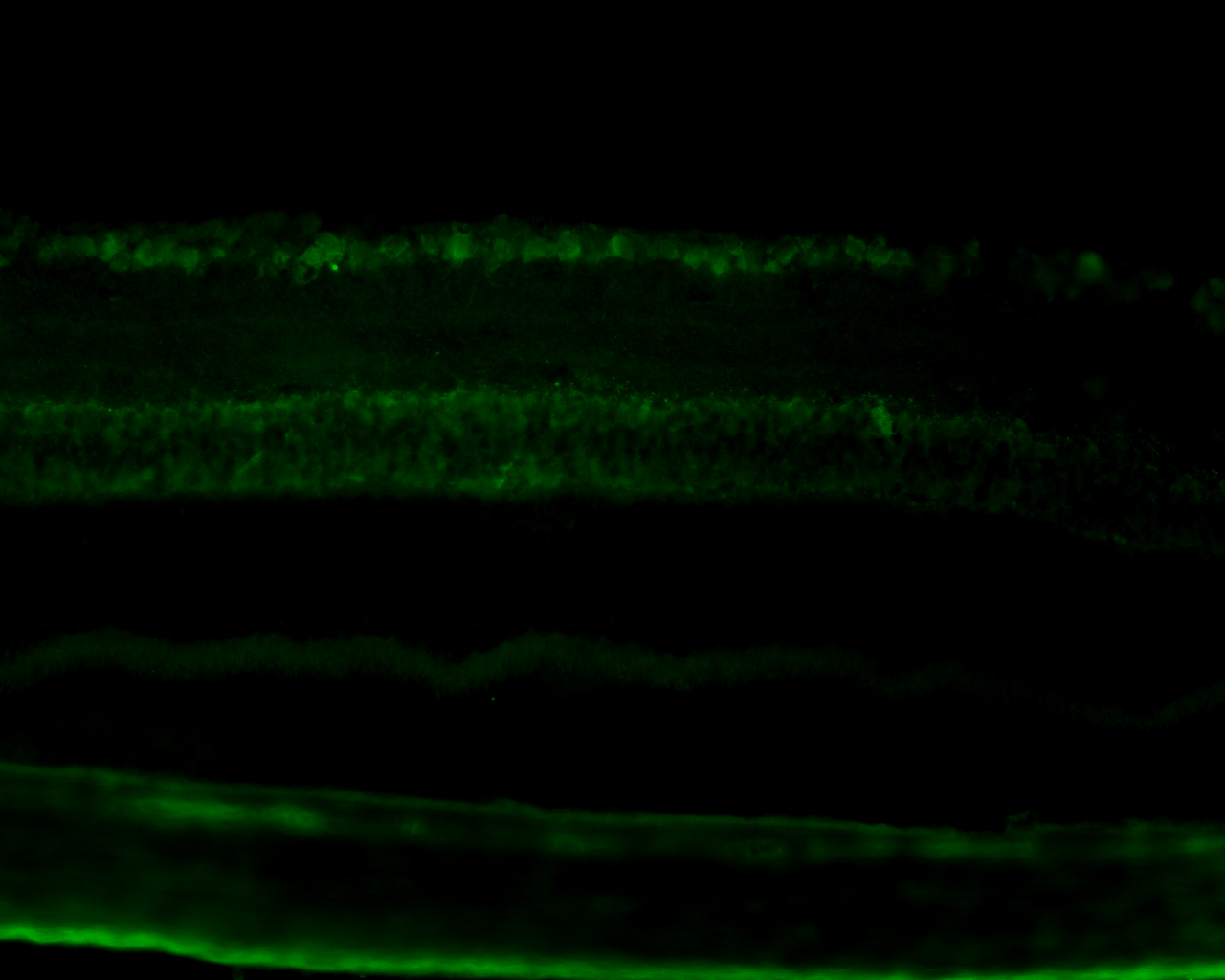

Supplement: S5 File — (ZIP) [file pone.0343169.s005.zip › S5 File/Fig1A_iASPP.tif]

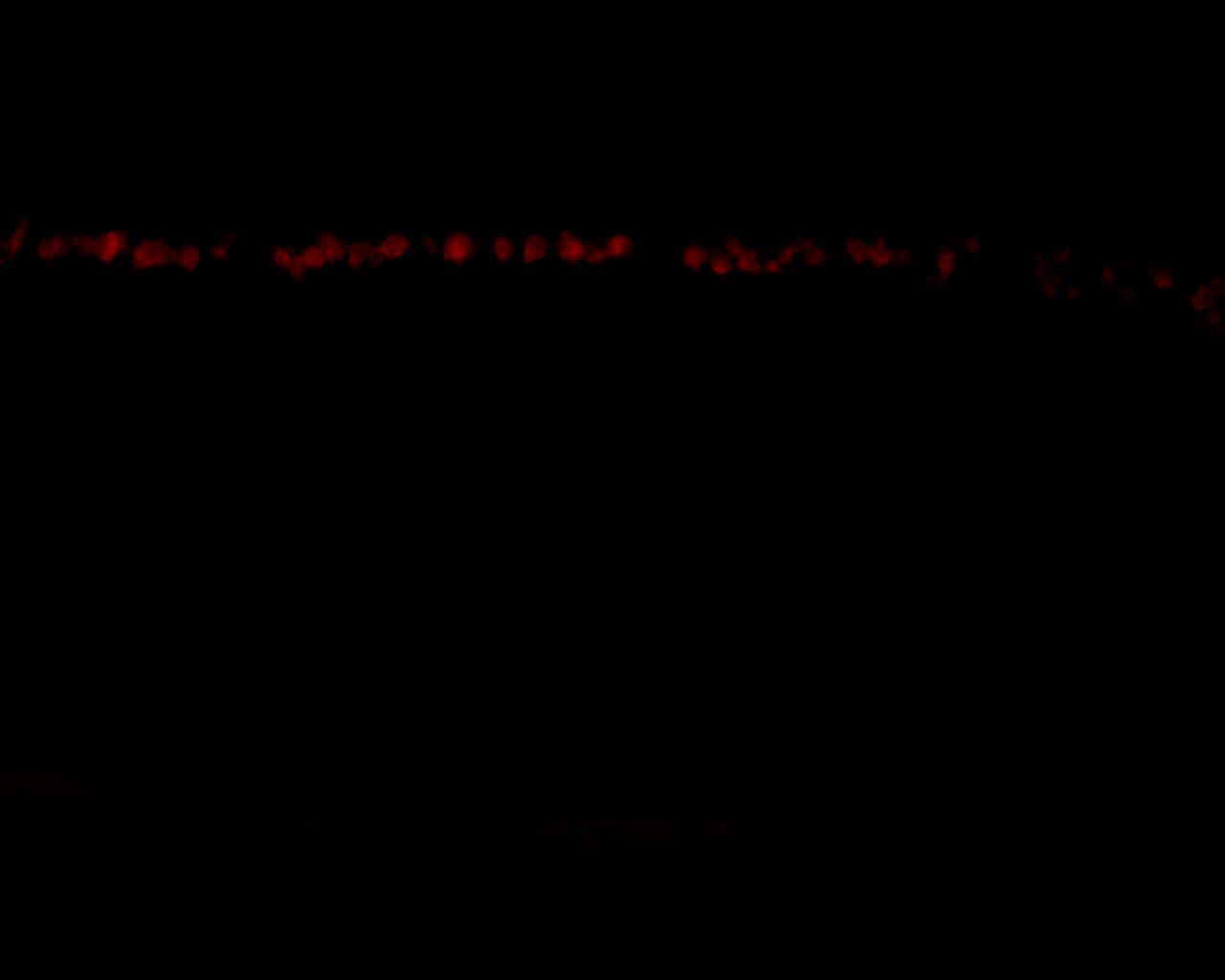

Supplement: S5 File — (ZIP) [file pone.0343169.s005.zip › S5 File/Fig1B_RBPMS.tif]
